# The digestive and defensive basis of carcass utilization by the burying beetle and its microbiota

**DOI:** 10.1038/ncomms15186

**Published:** 2017-05-09

**Authors:** Heiko Vogel, Shantanu P. Shukla, Tobias Engl, Benjamin Weiss, Rainer Fischer, Sandra Steiger, David G. Heckel, Martin Kaltenpoth, Andreas Vilcinskas

**Affiliations:** 1 Department of Entomology, Max Planck Institute for Chemical Ecology, D-07745 Jena, Germany; 2 Max Planck Institute for Chemical Ecology, Research Group Insect Symbiosis, D-07745 Jena, Germany; 3 Department for Evolutionary Ecology, Johannes Gutenberg University, D-55128 Mainz, Germany; 4 Fraunhofer Institute for Molecular Biology and Applied Ecology (IME), D-52074 Aachen, Germany; 5 University of Ulm, Institute of Evolutionary Ecology and Conservation Genomics, D-89081 Ulm, Germany; 6 Institute for Insect Biotechnology, Justus Liebig University of Giessen, D-35392 Giessen, Germany

## Abstract

Insects that use ephemeral resources must rapidly digest nutrients and simultaneously protect them from competitors. Here we use burying beetles (*Nicrophorus vespilloides*), which feed their offspring on vertebrate carrion, to investigate the digestive and defensive basis of carrion utilization. We characterize gene expression and microbiota composition in the gut, anal secretions, and on carcasses used by the beetles. We find a strict functional compartmentalization of the gut involving differential expression of immune effectors (antimicrobial peptides and lysozymes), as well as digestive and detoxifying enzymes. A distinct microbial community composed of Firmicutes, Proteobacteria and a clade of ascomycetous yeasts (genus *Yarrowia*) is present in larval and adult guts, and is transmitted to the carcass via anal secretions, where the yeasts express extracellular digestive enzymes and produce antimicrobial compounds. Our results provide evidence of potential metabolic cooperation between the host and its microbiota for digestion, detoxification and defence that extends from the beetle's gut to its nutritional resource.

## Abstract (web summary)

Burying beetles feed their offspring on the carrion of vertebrate animals. Here, the authors study gene expression in the insect's gut, as well as the composition of the microbiota in the gut and in carcasses, providing evidence for metabolic cooperation between host and specific microbes.

Interspecific interactions have important implications in shaping the dynamics of biological communities on ecological and evolutionary timescales. Species may respond to selection pressures arising from such interactions by combating antagonistic members, or forming mutualistic associations with others. Among these, the association between an animal host and its microbiota is increasingly recognized to fundamentally affect animal life by shaping host behaviour, development, immunity and metabolic homeostasis^1^. This is also true for insects, where ancient associations with microorganisms have enabled the colonization of novel niches by exploiting unusual diets and overcoming host defences^1^,^2^,^3^,^4^,^5^.

Insects feed on an extraordinary range of resources, some of which are highly unusual and challenging to consume. These foods may also be provisioned to their offspring through mass provisioning or progressive provisioning, requiring specialized behavioural and chemical strategies to prolong food availability, and may be associated with the evolution of parental care^6^. These strategies may originate from the insect, such as venom peptides in parasitoid wasps and hydrophobic secretions in beewolf wasps that preserve paralysed prey^7^,^8^. Alternatively, they can originate from symbionts, such as antibiotic-producing bacteria present in fungus-farming ants that defend mycelia of the cultivated fungus^9^. However, the mechanisms used to protect nutritional resources remain unknown in most insects, as does the mechanistic relationship with parental care behaviours such as the application of secretions or oral regurgitations to larvae. Furthermore, in the case of ephemeral and patchy resources that undergo rapid physicochemical and biotic changes, larvae are selected to maximize their growth rates and accelerate development^10^. Rapid growth requires the efficient digestion of nutrients to maximize nutrient assimilation and biomass conversion, along with robust antimicrobial defences to suppress microbial competitors. It remains unclear how the digestive and antimicrobial repertoires of insects that rely on ephemeral resources are partitioned between hosts and their microbiota, and whether these processes influence parental care behaviours. The carrion beetles (Coleoptera: Silphidae), which use small vertebrate carcasses as food for their larvae, provide suitable models to investigate these processes because they show larval food provisioning and also utilize a resource that is ephemeral and patchy^10^.

Carrion utilization involves intense interspecific competition. Burying beetles that use carcasses for breeding face two major challenges: the suppression of microbial growth to preserve carrion quality, and the rapid and efficient digestion of nutrients to maximize the rate of larval development. Microorganisms are under strong selection pressure to monopolize carcass nutrients by producing toxins or metabolites that render the meat unpalatable to competing vertebrates and invertebrates^11^. Unregulated microbial growth on carrion can deplete the nutrients, produce acids and gases, facilitate anaerobic conditions that favour toxin-producing bacteria such as *Clostridium* spp. and promote the accumulation of biogenic amines leading to nitrogen toxicity^12^ that can reduce beetle fitness^13^. Burying beetles therefore need robust antimicrobial defences to suppress microbial growth and/or mutualistic bacteria producing antimicrobial compounds that fulfill a similar role^14^. Furthermore, carrion has a narrow carbon-to-nitrogen ratio and a high water content^12^ and beetles therefore face competition with saprophytic microorganisms that use the carbohydrates, lipids (triglycerides) and proteins present in the carrion. Maximal larval growth rates require efficient digestive enzymes to assimilate carcass nutrients, or a community of mutualistic gut microbiota that augment the host enzymes or expand the diversity of compounds that can be utilized by the beetles^2^,^3^. It is not clear how burying beetles achieve both of these tasks, but it is evident that the carrion does not putrefy and its quality is successfully preserved despite being buried in the warm and moist environment of the soil.

Adult burying beetles discover and bury carcasses, lay eggs in nearby soil and the hatching larvae migrate to feed on the carcass. Preservation of the carcass is thought to be achieved by smearing the carcass with anal and oral secretions that have broad-spectrum antimicrobial activity against bacteria and fungi^15^. This behaviour likely increases larval survival^16^. The oral secretion, which is replenished over time on the carcass surface^17^, may also contain regurgitated or predigested food to feed early-instar larvae. The anal and oral secretions contain more than 30 different volatile^18^ and antimicrobial compounds^15^,^16^,^19^,^20^, some of which (including lysozyme) may protect the carrion and the larvae from microbial infection^18^,^19^,^21^. The lytic activity of the anal secretions is upregulated during the breeding cycle^16^,^21^, when the beetles defend the carcass and larvae against microbial colonization. The screening of immunity-related burying beetle genes identified at least four antimicrobial peptide (AMP) groups (the defensin, attacin, attacin-like and Cp1 families) and a number of other genes putatively involved in antimicrobial defence^22^. Transcriptomic analysis revealed several more lysozymes and AMPs including antifungal thaumatin peptides that are upregulated in breeding beetles in a context-dependent manner, when the beetles are defending and preserving carrion^19^,^20^,^21^. The oral secretions probably originate from the foregut and midgut, and anal secretions from the hindgut. The oral and anal secretions differ in their pH (ref. 18), and possibly in the composition of AMPs and the microbial community. However, little is known about the spatial distribution of different microbial species in the gut and their impact on the activity of the anal and oral burying beetle's secretions.

In this study, we focus on the carrion beetle *Nicrophorus vespilloides* Herbst and investigate the role of the beetle gut tissue and its microbiota in (1) the digestion of carrion, (2) the detoxification of microbial toxins on carrion and (3) the chemical preservation of carcass during breeding and protection from antagonists and competitors. To determine the relative contributions of the host and its microbiota, we carried out a combined analysis of the host gut transcriptome and the microbiome, and localized the antimicrobial peptides, putative digestive and detoxification enzymes, and different microbial species in different regions of the gut. We also isolated microbes from the beetles and compared their metabolites with those present in the anal and oral secretions. Finally, we analysed transcripts from the feeding cavity of the carcass (the portion of the carcass where larvae reside and feed) to determine which microbes were active on the carcass and to investigate their potential contribution to carcass utilization.

## Results

### Morphology of the *N. vespilloides* alimentary canal

**Figure 1:**
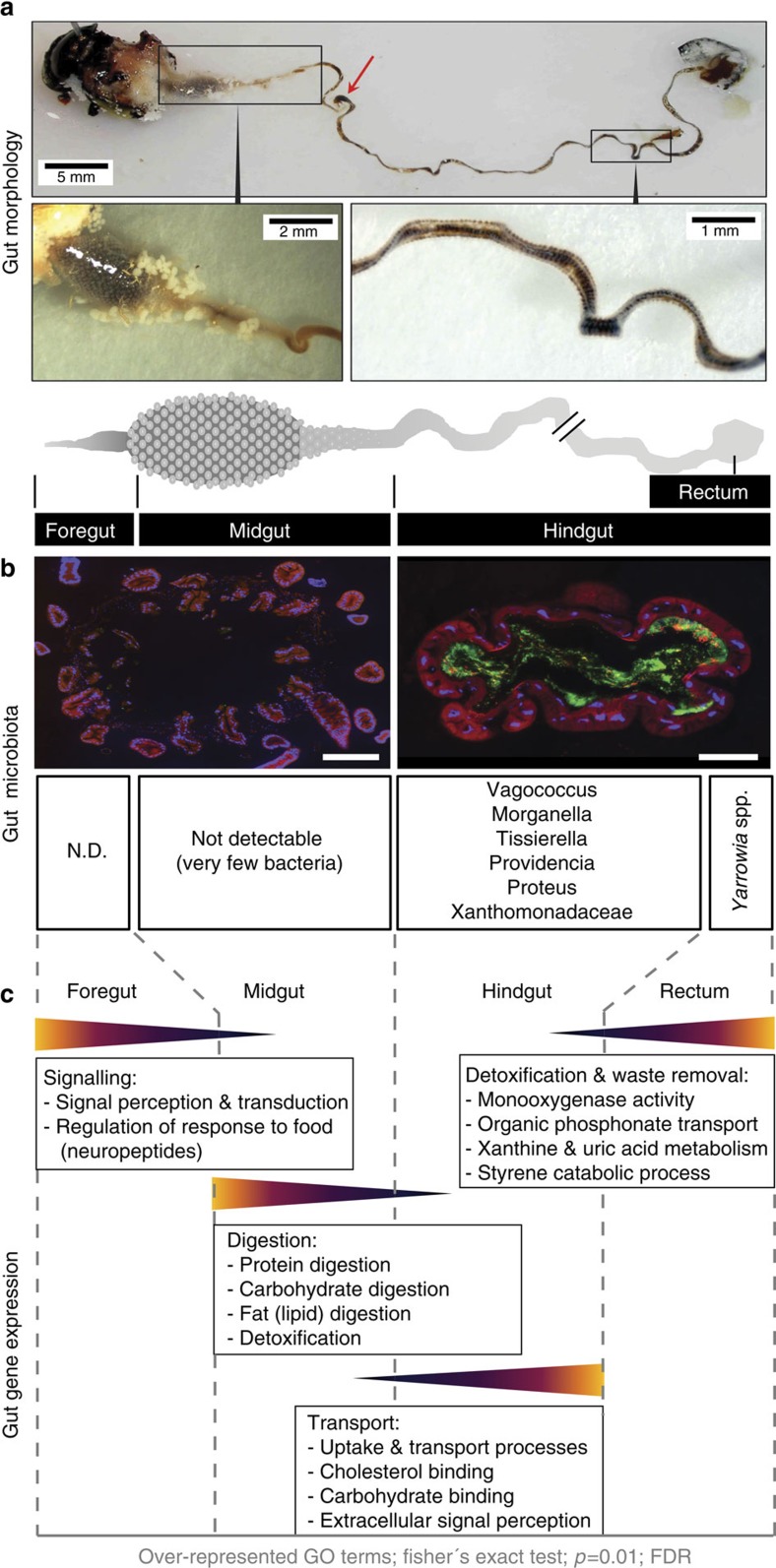
Gut transcriptome and gut microbial community of *N. vespilloides*. (**a**) Morphology of a male *N. vespillloides* gut showing the complete gut structure as well as midgut and flat hindgut region with bulbous inflations. The red arrow indicates the position where the gut was separated into the midgut and hindgut portions during dissection. (**b**) Fluorescence *in situ* hybridization revealed some sections of the midgut with low (not detectable) bacterial prevalence, but a bacterially abundant hindgut region (bacteria stained with the general bacterial probe (green) EUB338-Cy5). Scale bars, 200 μm. (**c**) Differential representation of GO terms, reflecting metabolic differences between gut regions, with the foregut and midgut involved in signalling and digestion, the hindgut involved in the transport of nutrients and the rectum involved in waste removal and detoxification.

We dissected both female and male adult *N. vespilloides* beetles to determine whether the gut is functionally compartmentalized and to delineate each area. In both sexes, the midgut is characterized by a voluminous region and a subsequent narrower region, both surrounded by numerous caeca, followed by a long, flat hindgut that ends in a widening rectum (Fig. 1a and Supplementary Fig. 1). Whereas the portion connecting the midgut to the hindgut has a circular cross-section, the much longer section that follows is flat and shows characteristic bulbous inflations. We divided the gut into midgut and hindgut portions according to the location of the Malpighian tubules and as shown in Fig. 1a. The region proximal to the voluminous midgut section was called the foregut. There were no obvious structural differences between female and male guts. We analysed gene expression and the composition of the microbiota in each of these gut regions separately.

### The *N. vespilloides* gut transcriptome

To determine whether the distinct morphological characteristics of the *N. vespilloides* gut sections also reflected differences in gene expression, we prepared poly(A)^+^ enriched mRNA from the foregut, midgut, hindgut and rectum and analysed the corresponding transcriptomes using Illumina HiSeq technology with paired-end (2 × 100 bp) reads. For *de novo* transcriptome assembly, we combined the RNA-sequencing (RNA-Seq) data from four gut samples as well as sequence data from adult heads, fat body and muscle tissues.

### Gut GO enrichment analysis

Fisher's exact test with a false discovery rate-corrected *P* value of <0.01 was used to identify the overrepresentation of Gene Ontology (GO) terms among lists of differentially expressed genes (eightfold change or higher) in the different gut section data sets relative to the complete reference data set that contained the assembly of all sequences. There were clear differences among the gut region-specific transcriptomes based on the abundance of GO terms (Fig. 1c and Supplementary Fig. 1). In the foregut, the enriched GO terms included G protein-coupled receptor signalling, sequence-dependent DNA binding, transcription (associated with signalling, homeostasis, signalling of perception and transduction) and neuropeptides (Fig. 1c and Supplementary Fig. 1). In the midgut, the enriched GO terms included protein and carbohydrate digestion, oxygen transport and detoxification. The hindgut was enriched for GO terms related to uptake and transport processes, carbohydrate binding and extracellular signal perception. The GO terms associated with the rectum suggested that this tissue is involved in the detoxification and removal of foreign compounds, xanthine and uric acid metabolism and styrene catabolic processes, among other functions (Fig. 1c).

### Gene expression related to digestion and detoxification

Based on the region-specific differences in gut gene expression, we identified functional categories related to neuropeptides, detoxification, digestive enzymes and transport processes, and visualized gene expression patterns across the four gut sections as well as in beetle heads, muscle and fat body tissues. The expression of several genes and functional categories was restricted to individual gut sections. For example, although genes related to neuropeptides, hormonal regulation and signal transduction were expressed in all gut regions, most of the genes were expressed at higher levels in the foregut (Supplementary Fig. 2). Enzymes involved in the digestion of macromolecules such as starch and proteins were expressed at high levels in the midgut and to a lesser extent in the foregut, and glucosidases, trypsins, chymotrypsins and carboxypeptidases were expressed at minimal levels if at all in the hindgut and rectum (Supplementary Figs 3 and 4). Similarly, many detoxification-related genes were expressed preferentially in the midgut, including uridine diphosphate (UDP)-glycosyltransferases, esterases and cytochrome *P*450s, but not glutathione *S*-transferases (Supplementary Figs 5 and 6). The hindgut was characterized by the strong expression of genes encoding sulfate, ammonia, trehalose and monocarboxylate transporters, as well as genes related to ammonia and amino acid metabolism and detoxification, reflecting its absorptive and homeostatic role (Supplementary Figs 5 and 7). The high-level expression of carbonic anhydrases may explain the basic pH of the hindgut determined previously^18^. Most genes associated with lipase activity, lipid and sterol binding, lipid modification and transport proteins such as apolipophorins, fatty-acyl reductases and vitellogenin, as well as small molecule binding proteins, were preferentially expressed in the rectum and only minimally expressed in other regions (Supplementary Figs 8 and 9).

### Gene expression related to innate immunity

In addition to digestion, detoxification, regulatory processes, nutrient transport and absorption, the *N. vespilloides* gut revealed evidence of regionally specific innate immunity. *De novo* transcriptome assembly and annotation identified a total of 27 putative AMPs and 13 lysozymes expressed in the fat body, foregut, midgut and hindgut, representing diverse families and functions. The diversity of AMPs was comparable to that observed in the whole-beetle *N. vespilloides* transcriptome^19^, comprising one cecropin, one KTX toxin-like peptide, five members of the attacin and coleoptericin families, five members of the defensin and defensin-like peptide families, six thaumatins, nine chicken-type (c-type) lysozymes and four invertebrate-type (i-type) lysozymes.

**Figure 2:**
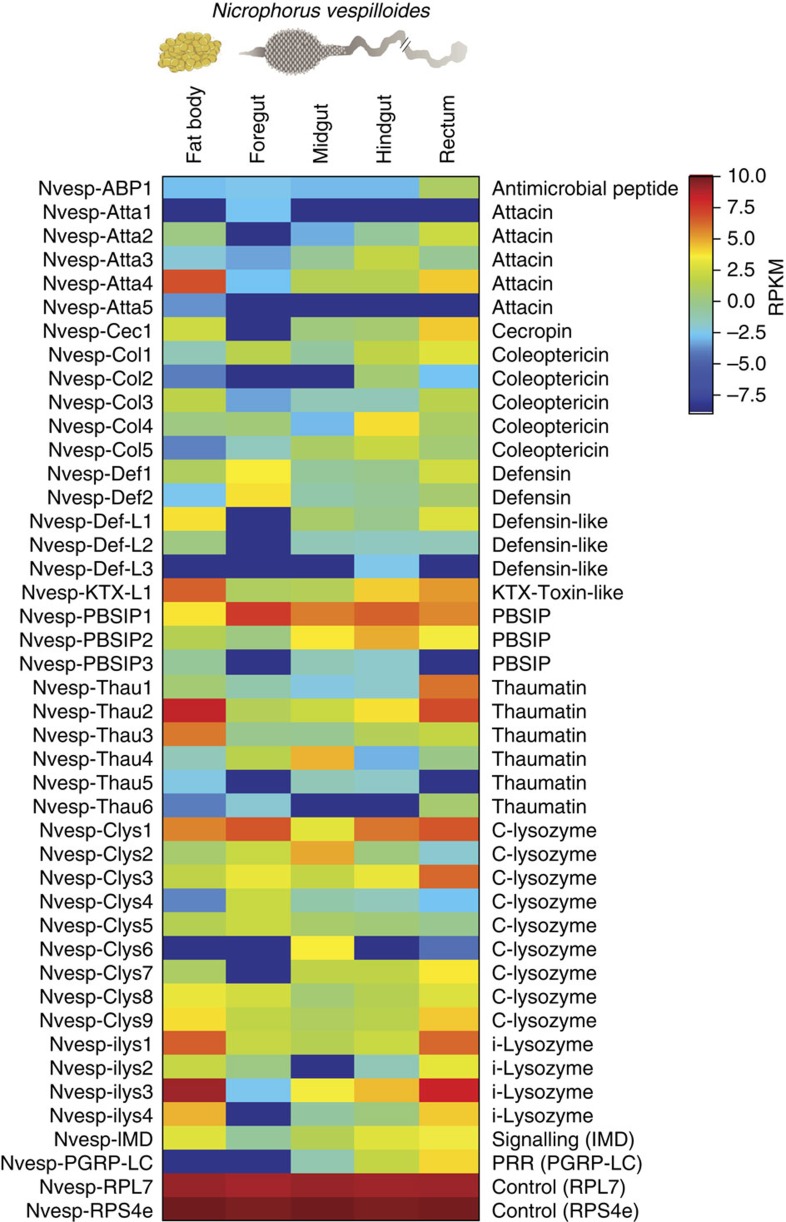
Relative gene expression levels of AMPs and lysozymes in *N. vespilloides* tissues. Different gut regions and the fat body of pooled *N. vespilloides* males and females were analysed. The housekeeping genes *RPL7* and *RPS4e* were used as controls to verify consistent expression patterns in the different tissues. Log2-transformed RPKM values are plotted, with warmer colours representing higher relative gene expression levels.

Quantitative analysis of putative transcripts based on reads per kilobase of contig per million mapped reads (RPKM) values revealed the differential expression of AMPs in different gut regions (Fig. 2). The rectum expressed the most diverse spectrum of AMPs and lysozymes, followed by the midgut and hindgut (Shannon's diversity index, Supplementary Table 1). The foregut expressed the highest levels of defensin-1, defensin-2 and PBSIP-1, whereas the midgut expressed the highest levels of c-lysozyme-2, c-lysozyme-6 and c-lysozyme-1. The hindgut expressed the highest levels of attacin-3, coleoptericin-4, coleoptericin-5, PBSIP-1 and PBSIP-2, whereas the rectum expressed the highest levels of attacins 2 and 4, coleoptericins 1 and 3, KTX toxin-like peptide, PBSIP-2, thaumatins 1–3 and 6, c-lysozymes 1, 3 and 7–9, and i-lysozymes 1–4. Several AMPs were exclusive to specific gut regions, for example, attacin-1 was exclusively expressed in the foregut, defensin-like-3 was exclusively expressed in the hindgut and c-lysozyme-6 was strongly expressed in the midgut but at low to undetectable levels in the other regions. The fat body was characterized by the strong expression of i-lysozymes 1, 3 and 4, thaumatin-2, KTX toxin-like peptide and attacin-4 (Fig. 2).

### The microbial community in the *N. vespilloides* gut

**Figure 3:**
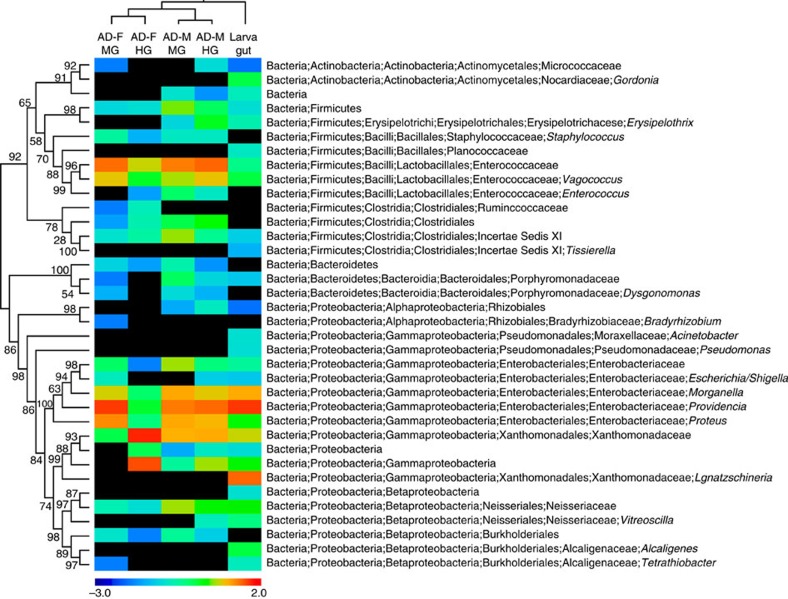
Bacterial community composition of the *N. vespilloides* gut. Composition of the bacterial community in the gut of *N. vespilloides* larvae and the midgut and hindgut of adult males and females was studied by TEFAP analysis of 16*S* rRNA gene sequences. The heat map shows the abundance of bacterial taxa (log-transformed), with warm colours indicating high abundance, cold colours indicating low abundance and black denoting absence. Taxonomy was assigned to representative sequences using the RDP classifier implemented in QIIME, and the phylogeny was generated in FastTree, based on an alignment produced using SINA and ARB. The samples were clustered based on the unweighted UniFrac distances of microbial community profiles (AD, adult; M, male; F, female; MG, midgut; HG, hindgut).

**Figure 4:**
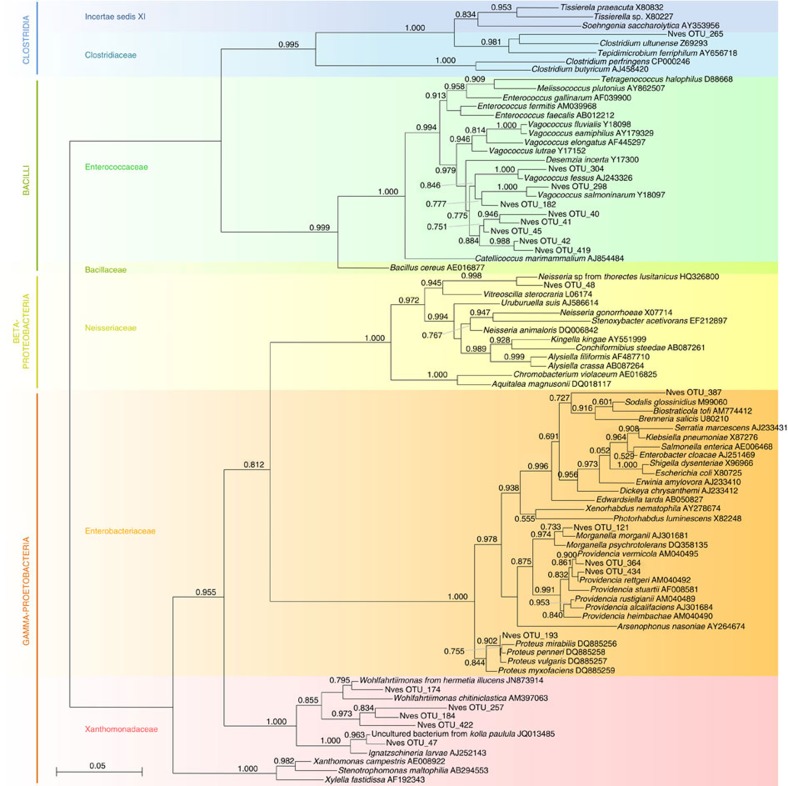
Phylogenetic affiliation of the 20 most abundant bacterial OTUs in adult and larval *N. vespilloides*. Based on partial 16*S* rRNA gene sequences (TEFAP). Sequences were aligned in SINA and ARB, and the phylogeny was reconstructed using FastTree.

To investigate the potential contribution of microbial symbionts to the utilization and defence of nutritional resources by *N. vespilloides*, we analysed the bacterial community in the guts of male and female adult beetles as well as anal secretions and larvae. Tag-encoded 454 FLX amplicon sequencing (TEFAP) yielded a total of 51,416 bacterial 16*S* ribosomal RNA (rRNA) gene sequences after trimming and quality control (Supplementary Data 1). Rarefaction analysis indicated that the microbial communities were sampled exhaustively (Supplementary Fig. 10). Male and female adult beetles showed qualitatively and quantitatively similar bacterial gut communities, which were dominated by obligate or facultative anaerobic Gammaproteobacteria and Firmicutes, with minor populations of Alphaprotebacteria and Betaproteobacteria, Bacteriodetes and Actinobacteria (Fig. 3). The adult gut bacterial communities revealed abundant operational taxonomic units (OTUs) associated with diverse bacterial taxa (Fig. 4) including *Morganella morganii*, *Proteus* spp. and *Providencia* spp. (all Gammaproteobacteria: Enterobacteriaceae), *Wohlfahrtiimonas chitiniclastica* (Gammaproteobacteria: Xanthomonadaceae), *Vagococcus* spp. (Firmicutes: Enterococcaceae), *Tissierella*/*Clostridium* spp. (Firmicutes: Incertae Sedis XI) and the family Neisseriaceae (Betaproteobacteria). These OTUs were also detected in the larval stages at varying relative abundances, in addition to highly abundant *Ignatzschineria larvae* (Gammaproteobacteria: Xanthomonadaceae) (Figs 3 and 4 and Supplementary Data 1). We confirmed the presence of these bacteria by diagnostic PCR using taxon-specific 16S primers, and detected all of the most abundant OTUs (except *M. morganii*) in 100% of the larvae and 62–100% of the adults we tested (Supplementary Table 4), indicating that the bacterial community was consistent across individual beetles. In comparison with adult and larval beetle guts, the bacterial community in adult *N. vespilloides* anal secretions was shifted in favour of the Firmicutes (Supplementary Fig. 11), with a highly abundant population of *Vagococcus* (43.7% of the bacterial community). Although the Xanthomonadaceae (2.04%) and Clostridiales (17.6%) were frequently detected, genera representing the Enterobacteriaceae (for example, *Providencia* and *Morganella*) were absent from anal secretions (Supplementary Data 1 and Supplementary Fig. 11).

**Figure 5:**
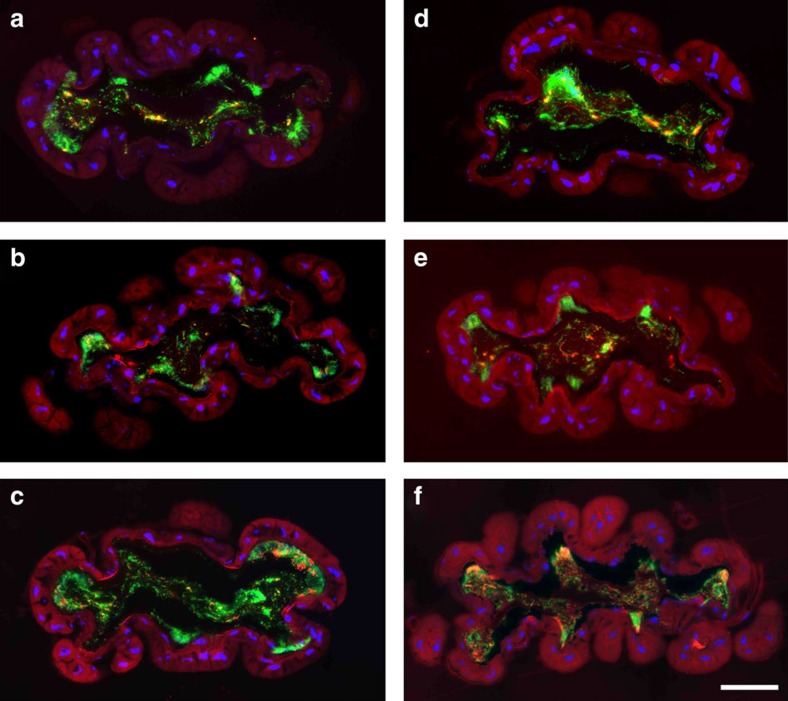
Localization of major bacterial taxa in the hindgut of an *N. vespilloides* adult female by fluorescence *in situ* hybridization. Bacteria were stained with the general bacterial probe EUB338-Cy5 (green) and taxon-specific Cy3-labelled oligonucleotide probes (red). Nuclei were counterstained with 4,6-diamidino-2-phenylindole (DAPI; blue). (**a**) *Vagococcus*, (**b**) *Tissierella*, (**c**) Xanthomonadaceae, (**d**) *Morganella*, (**e**) *Providencia* and (**f**) *Proteus*. Scale bar, 100 μm.

Six of the seven most dominant bacterial taxa (*Morganella*, *Proteus*, *Providencia*, Xanthomonadaceae, *Vagococcus* and *Tissierella*, but not Neisseriaceae) were localized by fluorescence *in situ* hybridization in the hindgut of adult beetles (Fig. 5). The bacteria were localized within the lumen or associated with the epithelium of the flat hindgut portion, without any apparent niche separation among the bacterial taxa. Surprisingly, major portions of the midgut were completely devoid of any bacteria in three out of four adult beetles (Supplementary Fig. 12a), revealing the sanitization of at least some sections of the midgut by the adults. The fourth individual contained large numbers of *Vagococcus* as well as some *Providencia*, but none of the other major taxa were detected in the midgut (Supplementary Fig. 12b).

**Figure 6:**
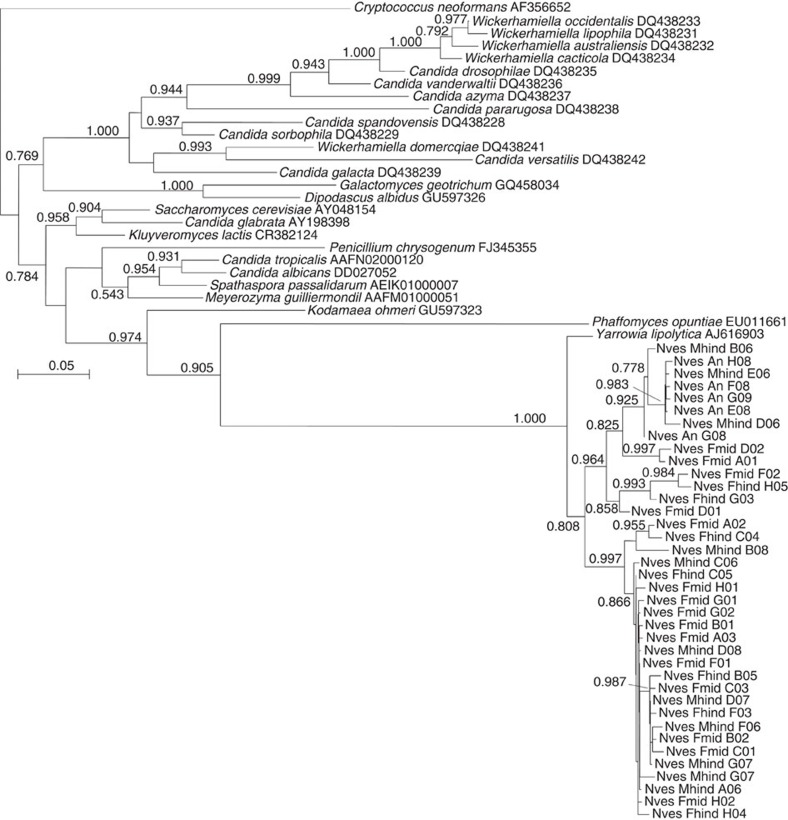
Phylogenetic affiliation of representative fungal LSU rRNA gene sequences obtained by cloning and sequencing amplicons from adult male and female beetle guts. Sequences likely representing a new yeast species related to *Yarrowia lipolytica* were abundantly associated with *N. vespilloides* gut tissues. Sequences were aligned in SINA and ARB, and the phylogeny was reconstructed using FastTree.

The fungal communities of the midgut and hindgut in males and females were characterized using both traditional PCR-based cloning and sequencing of the partial large subunit (LSU) rRNA gene and by pyrosequencing portions of the small subunit (SSU)–LSU rRNA internal transcribed spacer (ITS). All 53 cloned LSU sequences were most closely related to the biotechnologically important yeast *Yarrowia lipolytica* (Ascomycota: Dipodascaceae), and the majority of the TEFAP ITS sequences were also taxonomically assigned to *Yarrowia* (Table 1 and Supplementary Table 2). The remaining ITS sequences originated from diverse and inconsistently detected Ascomycota and Basidiomycota. Diagnostic PCRs confirmed the presence of *Yarrowia* in seven out of eight tested adults and in all six tested larvae (Supplementary Table 2). Phylogenetic analysis of the *Yarrowia* LSU sequences indicated that a closely related community of *Yarrowia* strains colonized the beetle gut (Fig. 6), all of which formed a monophyletic sister clade to the *Y. lipolytica* type strain. These yeasts were frequently found at a high density in the rectum of the adult beetles (Supplementary Fig. 13).

The cloning and sequencing of fungal LSU amplicons also revealed the presence of yeasts related to *Y. lipolytica* in the anal secretions of adult beetles (Fig. 6). This was confirmed by the culture-dependent characterization of anal secretions, from which a total of 91 colonies were isolated using the pooled anal secretions of adult females. Comparison of the 900 bp 28S rRNA fragment amplified by the LS1/LR5 fungal primers confirmed that 85 of these colonies were highly similar (up to 99% identity, nucleotide BLAST using the National Center for Biotechnology Information (NCBI) non-redundant (nr) database) to the Y*arrowia* yeasts characterized in the adult beetle gut^14^. Three remaining colonies isolated from the anal secretions were found to represent *Debaryomyces hansenii* (99% identity), whereas two isolated colonies could not be identified. As observed in the midgut and hindgut, we also found strain diversity among the *Yarrowia* isolates from the anal secretions. The isolates formed two distinct clades (clades I and II, Supplementary Fig. 14) within the partial 28S phylogeny, probably representing at least two different species within the genus *Yarrowia*. Clade I comprised four isolates (strains C09, C11, E12 and E02), whereas clade II comprised the remaining 81 *Yarrowia* isolates (Supplementary Fig. 14). *Yarrowia*-like strains from these clades also differed in their colony morphology as demonstrated by the two representative strains B02 and C11, representing clades I and II, respectively (Supplementary Fig. 14). No *Yarrowia*-like yeast strains could be detected in the soil in which the beetles were reared, on the surface of mouse carcasses before they were offered to the beetles or from the internal organs of the same carcasses (Supplementary Fig. 15) using the *Yarrowia*-specific primers 28S-fwd2/28S-rev4 (ref. 14). Therefore, we excluded the possibility that the *Yarrowia* detected in the beetle gut and anal secretions could be transient or ‘contaminants' acquired from the soil or the carcass.

### Transcriptomic analysis of carcasses utilized by *N. vespilloides*

**Figure 7:**
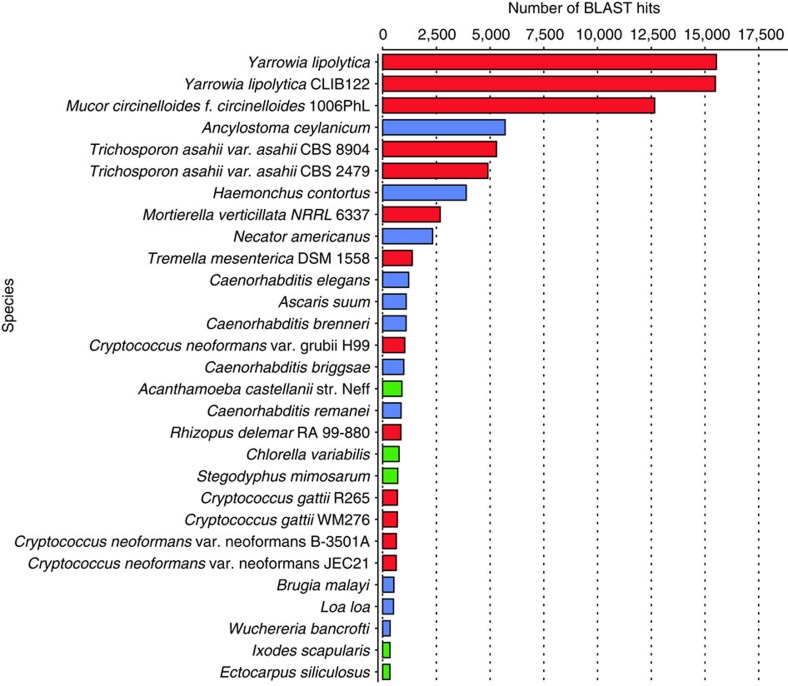
Biological community distribution based on top hits of RNA transcripts found on carcasses used by *N. vespilloides*. Fungal taxa are shown in red, nematodes in blue and others as green bars.

The diversity and abundance of the biological community present on the breeding carcasses were determined by sequencing pooled RNA isolated from the feeding cavity of two carcasses prepared by breeding beetles and utilized by *N. vespilloides* larvae. The carcass was exposed to the beetles for ∼7 days, during which it was buried and a feeding cavity was created by the beetles. The resulting Illumina sequences were assembled *de novo* and the transcriptome was annotated using BLAST and Blast2Go software. The most abundant eukaryotic transcripts detected in the feeding cavity were *Yarrowia* sequences, but other fungal transcripts included those from the genera *Mucor* (Zygomycota: Mucorales), *Trichosporon* (Basidiomycota: Tremellales), *Mortierella* (Zygomycota: Mortierellales), *Cryptococcus* (Basidiomycota: Tremellales) and *Rhizopus* (Zygomycota: Mucorales) (Fig. 7). *Trichosporon* and *Cryptococcus* were also detected in the adult midgut or hindgut, but neither was consistently present in both males and females (Supplementary Table 3). We also detected carcass transcripts belonging to several nematodes, including *Ancyclostoma* (Nematoda: Rhabditida) and *Haemonchus* (Nematoda: Rhabditida) (Fig. 7). Because *Yarrowia* transcripts were the most abundant in the carcass cavity, and *Yarrowia* were also abundant in the hindgut/rectum and anal secretions, we focused our analysis on the *Yarrowia* transcripts to determine the potential functions of the yeasts colonizing the carcasses. BLAST analysis of the 98,801 unique transcripts detected on the carcass surface revealed 15,586 transcripts with the greatest similarity to *Y. lipolytica* genes (Fig. 7). GO assignments of the contigs with best BLAST hits against *Y. lipolytica* confirmed that a broad range of molecular functions and biological processes are covered (Supplementary Fig. 16). The KEGG-KAAS annotation pipeline indicated that many of these transcripts were associated with carbohydrate metabolism, lipid metabolism (including fatty acid synthesis and degradation), amino acid metabolism (including the synthesis of essential amino acids), vitamin and cofactor metabolism (Supplementary Table 6) or with glycerolipid and terpenoid biosynthesis. In many cases, most or all of the enzymes in each pathway were represented in the transcriptome data set.

The potential role of *Yarrowia* in carcass digestion was tested by screening the *Yarrowia* transcripts for the presence of sequences encoding extracellular lipases, proteases and creatinases. As shown in Supplementary Table 7, we detected transcripts for *Y. lipolytica* extracellular proteases, the extracellular enzyme triacylglycerol lipase (enzyme classification (EC) 3.1.1.3), enzymes involved in fatty acid degradation such as long-chain acyl-CoA synthetase (EC 6.2.1.3) and enzymes involved in fatty acid β-oxidation, indicating that *Yarrowia* on the carcass likely facilitates both protein and lipid metabolism. No transcripts representing extracellular creatinases were detected. We also detected *Yarrowia* transcripts encoding enzymes involved in sterol biosynthesis, converting farnesyl diphosphate (an intermediate in the sterol biosynthesis pathway) to lanosterol, fecosterol and ergosterol.

### Metabolic analysis of *Yarrowia* isolated from anal secretions

The potential role of *Yarrowia* in carcass preservation and digestion was investigated by analysing the extracellular metabolites produced by the two representative *Yarrowia* strains isolated from *N. vespilloides* anal secretions (B02 and C11, representing clades I and II, respectively) under *in vitro* conditions (Supplementary Fig. 17a). Using gas chromatography–mass spectrometry (GC–MS), we identified four low-molecular-weight extracellular metabolites produced by *Yarrowia* spp. that are also present in the anal secretions^18^: tetradecanoic acid (myristic acid) was produced in liquid medium, whereas octadecanoic acid (stearic acid), octadecenoic acid (oleic acid) and hexadecanoic acid (palmitic acid) were produced at high concentrations by both *Yarrowia* strains on solid medium. In addition, other low-molecular-weight compounds (such as aldehydes, esters of fatty acids and sterols) were also detected at different concentrations in the solid and liquid media (Supplementary Fig. 17b).

## Discussion

Insects that use ephemeral and sporadic resources minimize larval development times to avoid competition and predation^10^. A highly efficient digestive mechanism is required by such insects to support rapid nutrient assimilation and biomass conversion during larval development. At the same time, insects that provision food for their larvae must evolve behavioural and chemical food-preservation strategies that suppress competitors^6^. Burying beetles show both of these traits, that is, they utilize a highly nutritious and ephemeral resource (carrion) that they digest in a relatively short period of time, and they bury the carcass and chemically preserve its nutrients by applying oral and anal secretions^17^. Here, we show that both traits are supported by a metabolically rich gut transcriptome producing a diverse set of digestive enzymes, AMPs and lysozymes that may help to digest and preserve the carcass surface, as well as a consistent bacterial and fungal gut microbiota that is transmitted to the larvae through anal secretions applied to the carcass surface.

We identified AMPs and lysozymes that are differentially expressed in different regions of the gut, corresponding to their presumed or confirmed presence in anal and oral secretions. The expression of several of the AMPs, which are upregulated in breeding beetles^19^, was higher in the midgut or foregut, while others were expressed at the highest levels in the foregut and rectum (Fig. 2), suggesting they may also be present in both anal and oral secretions. The oral secretions are also present in regurgitated meals fed to early-instar larvae, and hence, together with anal secretions, they could sanitize the carcass as well as contribute to larval immunity and digestion. The lysozyme complexes probably evolved for release through *N. vespilloides* anal secretions and diversified to produce multiple enzyme variants in this species. The c-type and i-type lysozymes expressed in the *N. vespilloides* gut could show either digestive or immunity-related functions, the latter by potentiating the activity of other AMPs such as coleoptericins^23^,^24^. The diversity of AMPs and lysozymes may also protect against rapidly evolving pathogens or unpredictable pathogen diversity in the soil^25^,^26^. A potent cocktail of cecropin, defensin, thaumatin and attacin peptides was also detected in the gut transcriptome, providing a means to target bacteria, fungi and viruses by penetrating lipid membranes^27^. Interactions among diverse AMPs involving synergism, potentiation or functional diversification may enhance their antimicrobial effects, allowing more effective regulation of gut microbiota and sanitization of carcass surfaces^28^.

The high expression levels of two lysozymes and a thaumatin in the midgut and foregut could provide a first line of defence against food-associated entomopathogens, and could also sanitize the midgut environment to control the passage of microbes that eventually colonize the hindgut^29^. It is necessary to control the hindgut community because excretions are applied to carcasses, and dysregulation of the hindgut community could facilitate the transmission of pathogens and parasites to the carcass and larvae through anal secretions^30^, in addition to causing harmful effects in the adult beetle itself. Burying beetles may therefore be under strong selection pressure to regulate their hindgut/rectum microbial community that also determines the microbial composition of the anal secretions and the carcass. Such regulation could be achieved by regionally specific differential AMP and lysozyme expression, or by filtering out opportunistic microorganisms at the midgut level^31^. However, not all microbes are necessarily affected by lysozymes and AMPs, and closely associated microbiota, such as *Yarrowia*-like yeasts, appear to have adapted to the specific host environment *N. vespilloides* provides. The diversity of AMPs and lysozymes, their differential expression in the *N. vespilloides* gut and their possible involvement in carcass sanitization, regulation of gut microbiota and/or the supplementation of digestive enzymes highlights the evolutionary plasticity of *N. vespilloides* immune responses in relation to context-specific environments such as the presence of carcass and offspring.

The differential enrichment of GO terms in the *N. vespilloides* gut transcriptomes suggests that region-specific metabolic processes may contribute to meat digestion, nutrient transport and detoxification (Fig. 1). Midgut-specific gene expression is clearly dominated by proteins related to the breakdown of carbohydrates and the initial steps of protein digestion, suggesting that at least the initial steps of most digestive processes may take place in this section of the *N. vespilloides* gut. In the hindgut, we detected metabolic signatures for protein digestion and recycling, for example, xanthine metabolism and serine endopeptidases, indicating a high nitrogen turnover. The highly elongated hindgut may enable the efficient assimilation of nutrients, the processing of dry food particles or the prolonged digestion of refractory substrates. This can be achieved by providing more time and a greater surface area for the assimilation and processing of protein-rich carcasses. This is further supported by the hindgut-specific expression of many transporters, with roles in not only sulfate, carbohydrate, lactate and ketone transport, but also in the transport and metabolism of essential amino acids. In addition, some of these highly expressed genes, such as the monocarboxylate transporters and ornithine aminotransferases, are likely to play a role in the elimination of potentially toxic products, such as nitrogenous waste and organic anions resulting from the metabolism of large amounts of protein from the consumed carcass.

Although the rectum appears to be an inflated terminal part of the hindgut, the gene expression patterns in each region are distinct. The genes most strongly expressed in the rectum include those related to vitamin transport, lipid binding and metabolism, indicating that these processes predominantly take place in the short rectum and not in the longer hindgut. The highly specific expression of genes encoding α-tocopherol transfer proteins suggests the rectum plays an important role in the control of vitamin E, a key antioxidant. Furthermore, the strong expression of genes encoding small molecule binding proteins, including vitellogenins and carriers of lipids, phosphates, metals and hormones, suggests that the rectum also regulates the homeostasis of such molecules. Vitellogenins also act as pathogen pattern recognition receptors and as carriers of immune-priming signals^32^,^33^, suggesting a role in microbial recognition and regulation of the host immune response to the abundant microbial inhabitants in the *Nicrophorus* rectum.

The *N. vespilloides* gut also hosts a specialized microbial community, similar to that found in other flesh-eating insects such as sarcophagid flies^34^. Many of the hindgut bacteria were also present in anal secretions, and when smeared on carcasses they may facilitate preservation or digestion by producing antimicrobials or extracellular enzymes, respectively. OTUs representing the Xanthomonadaceae in the hindgut were related to the aerobic *I. larvae* isolated from sarcophagid flies and hematophagous mites^34^,^35^, and these have been detected in other Silphidae species^14^. Such microbes could promote carcass digestion by producing esterase, lipase and urease activities in the hindgut^36^,^37^. The obligate or facultative anaerobic Firmicutes (*Tissierella*, *Clostridiales* and *Vagococcus*) detected in adult and larval guts and anal secretions may facilitate meat digestion, because some species within this genus can digest creatine^38^. This is an energy-rich nitrogenous organic acid present in vertebrate muscles and organs, but it cannot be degraded by insects, and hence bacterial enzymes are necessary for efficient utilization. The Enterobacteriaceae (*Morganella*, *Providencia* and *Proteus*) have been detected in flesh flies^39^, and their function in *N. vespilloides* may involve the provision of urease activity or bacteriolytic enzymes, but they could also be pathogens^40^. The combined analysis of GO terms and microbial diversity suggests that beetles and their associated microbiota may complement each other to achieve carrion digestion and detoxification in the midgut, hindgut and rectum, thus enabling both the internal and external utilization of carrion.

The fungal community in the *N. vespilloides* gut and anal secretions was dominated by ascomycetous yeasts closely related to the biotechnologically important *Y. lipolytica* that can break down diverse carbon sources including hydrocarbons and lipids^41^. Their prevalence in adults, larvae, anal secretions and on the carcass surface suggests transmission from parent to offspring via the anal secretions. The prevalence of *Yarrowia* transcripts on the beetle-utilized carcass surface (Fig. 7) confirmed that the yeasts are metabolically active on the carcass and are resident rather than transient members of the beetle microbiota and the carcass microbial community. Their presence in anal secretions and on the carcass surface also suggests potential horizontal transmission to larvae through the carcass surface. The abundance of *Yarrowia* in anal secretions, which are applied from the early stages of carcass preparation, could lead to the early colonization of the carcass, offering a mechanism to prevent colonization by undesirable fungi during the breeding cycle. We ruled out the possibility of environmental acquisition of *Yarrowia* by adult beetles because the yeast was not detected in the soil, on the surface of mouse cadavers provided to the beetles or in the internal organs of the same cadavers (Supplementary Fig. 15). *Yarrowia* are also known to be present in virgin adults that had never had access to carcasses^14^, indicating that beetles must already possess the yeast at the time of their eclosion. We conclude that *Yarrowia* is most likely to be symbiont of *N. vespilloides* rather than a pathogen or parasite, based on the following observations. First, *Yarrowia* was present during all beetle life stages, as well as in the anal secretions and on the carcass surface, where it was also the most metabolically active microbe. Second, phylogenetic analysis indicated that these *Yarrowia* yeasts are monophyletic with *Y. lipolytica*, which is not known to be entomopathogenic, and its 18*S* sequence has diverged substantially from many known pathogenic *Candida* species^42^. The functional analysis of *Yarrowia* transcripts revealed their potential involvement in the digestion of carcass nutrients, but it is unclear whether these processes affect digestion, nutrient acquisition and beetle fitness. The *Yarrowia* metabolic processes included sterol biosynthesis and fatty acid metabolism on the carcass, as well as a number of secreted proteases, but we found no evidence for the presence of creatinases. The sterol biosynthesis pathway produces ergosterol, the major component of fungal cell membranes, and fatty acid metabolism converts fatty acids to acetyl-CoA and produces energy. The burying beetle could benefit from these pathways because insects cannot synthesize the sterol precursors needed to form lipid biostructures, steroid hormones and developmental regulators^2^,^43^,^44^. Yeasts often secrete sterols^45^ and if *Yarrowia* secrete essential sterol precursors in the rectum or on the carcass, these molecules could be utilized by the host, as previously suggested for fungal symbionts in other beetles and in leafcutter ants^46^,^47^,^48^. The role of these potential symbionts in sterol production in the rectum is supported by the finding that both sterol modifying enzymes (for example, sterol reductase) and sterol transport proteins (lipophorins) are expressed at high levels specifically in the host rectum (Supplementary Fig. 8). Similarly, the secretion of extracellular lipases on the carcass by *Yarrowia*, and fatty acid degradation, could supply the beetles with simple fatty acids through the digestion of triglycerides in the carrion. This activity is further supported by the finding that the *Yarrowia* isolates produced myristic acid, stearic acid and oleic acid on solid medium and glucose-containing liquid medium (Supplementary Fig. 15). Oleic acid is required for the activity of *Y. lipolytica* extracellular lipases^49^, and could thus facilitate extracellular lipid digestion in the carrion. These three fatty acids were also detected in the anal secretions^18^ and are known to possess antimicrobial activity^50^,^51^, and hence they could be involved in carcass preservation. Interestingly, these fatty acids have little or no inhibitory activity against yeasts, but strongly inhibit other fungi and bacteria^50^,^52^, indicating that certain *N. vespilloides* defences could be safe for *Yarrowia* but effective against other bacterial competitors.

The *N. vespilloides* transcriptome and microbiome together shed light on the potential mechanisms by which insects preserve and provision food for their larvae in competitive and pathogen-rich environments, yet at the same time maximize larval growth rates by digesting and assimilating the required nutrients. The differential expression of host digestive proteases and lipases, AMPs and lysozymes, and the characteristic bacterial and fungal microbiota in these species, may underlie basic parental behaviours such as the transfer of oral regurgitates containing enzymes, antimicrobials and symbionts, and the chemical sanitization of food resources. Such behaviours are common across many insects exhibiting parental care, and may therefore serve similar functions for digestion, detoxification and preservation as in *Nicrophorus*. These patterns in *N. vespilloides* also demonstrate different levels of task partitioning, one between the beetles and their microbiota, and the other between internal and external aspects of substrate utilization such as detoxification, microbial control and digestion in the gut and on the carcass. Our results indicate that metabolic cooperation with a phylogenetically and metabolically diverse microbial community of bacteria and yeasts may underlie the ecological success of *N. vespilloides*. Our results also support recent concepts in biology that distinguish between a flexible, environmentally modulated microbial pool, and a host-adapted core microbiota, differing in constraints on their maintenance and in their contributions to host adaptation^53^.

## Methods

### Insects

Parasite-free *N*. *vespilloides* male and female beetles (that is, not visibly infested with mites and nematodes) were provided by Josef K. Müller and Wolf Haberer, University of Freiburg, Germany. In addition to the standard laboratory culture, we have also collected beetles in the wild for subsequent gut microbiota analyses (see below for details). Beetles were cultured using ‘foster parents' as previously described^54^. Groups of up to six adults of the same sex were kept in transparent plastic containers (100 × 100 × 65 mm; Famos-Westmark, Lennestadt-Elspe, Germany) filled two-thirds with peat, in a 20 °C controlled-temperature room with a 16 h light cycle and 65% relative humidity. Each beetle was fed with one freshly decapitated mealworm (*Tenebrio molitor*) twice a week that were purchased from a commercial supplier (b.t.b.e. Insektenzucht GmbH, Germany). To facilitate breeding, each male–female pair was provided with a (thawed) mouse (*Mus musculus*) carcass (b.t.b.e. Insektenzucht GmbH, Germany) ∼4 h before the onset of darkness. Parents were removed 5 days after the hatching of their larvae. Containers were transferred to darkness after third-instar larvae appeared on the soil surface for pupation.

### RNA-Seq and *de novo* assembly

Total RNA was isolated from the head, muscle, fat body, foregut, midgut, hindgut and rectum tissue of two groups (replicates) of two male and female beetles (sexually mature, breeding adults, pooled for analysis). Each sample was transferred directly into tissue lysis buffer (InnuPREP RNA Mini Kit, Analytik Jena AG, Jena, Germany) and shock frozen at −80 °C before RNA isolation using the same kit. We also isolated RNA from two mouse carcasses prepared by two separate pairs of breeding *N. vespilloides* adults to investigate the prevalence of RNA transcripts in the feeding cavity of prepared carcasses. The carcass was exposed to the beetles for ∼7 days, during which it was buried and a feeding cavity was created by the beetles. The carcass cavity was swabbed with sterile, DNA-free cotton swabs (Sarstedt, Nümbrecht, Germany). At the time of swabbing, both the breeding male and female were present near the carcass and the feeding cavity contained actively feeding second-instar larvae. The RNA was extracted independently from two carcass samples using TRIsure (Bioline, Berlin-Brandenburg, Germany) according to the manufacturer's instructions, followed by DNase treatment (Turbo DNase, Thermo Fisher Scientific, Waltham, MA, USA) and was further purified using RNA Clean and Concentrator 5 (Zymo Research, Irvine, CA, USA). The integrity of all RNA samples was verified using an Agilent 2100 Bioanalyzer and an RNA 6000 Nano Kit (Agilent Technologies, Palo Alto, CA, USA). The quantity of RNA was determined using a Nanodrop ND-1000 ultraviolet–visible spectrophotometer (Thermo Fisher Scientific). The transcriptomes of the different adult tissue RNA samples were sequenced using poly(A)+ enriched mRNA fragmented to an average of 160 nucleotides. Gut and fat body samples were sequenced by the Max Planck Genome Center Cologne (MPGCC) on an Illumina HiSeq2500 Genome Analyzer platform, using paired-end (2 × 100 bp) reads. This yielded ∼30 million reads for each *N. vespilloides* tissue sample. Carcass-derived RNA transcripts were sequenced by GATC Biotech (Konstanz, Germany) on an Illumina HiSeq2500 Genome Analyzer platform, with the rapid Run Mode generating 20 million paired-end (2 × 125 bp) reads. Quality control measures, including the filtering of high-quality reads based on the score given in fastq files, removal of reads containing primer/adapter sequences and trimming of read length, were carried out using CLC Genomics Workbench v8.1 (http://www.clcbio.com). The *de novo* transcriptome assemblies of the beetle and carcass samples were prepared using the same software, by combining all seven *N. vespilloides* tissue samples or the single carcass sample, respectively, and selecting the presumed optimal consensus transcriptome as previously described^55^. The resulting final *de novo* reference transcriptome assembly (backbone) of *N. vespilloides* contained 55,918 contigs (minimum contig size=200 bp) with a N50 contig size of 1,271 bp and a maximum contig length of 24,850 bp. The final *de novo* transcriptome assembly of the carcass sample contained 98,769 contigs. The resulting sequences were used to search the NCBI nr nucleotide database with the blastall program. Homology searches (BLASTx and BLASTn) and functional annotation according to GO terms (http://www.geneontology.org), InterPro terms (InterProScan, EBI), EC codes, and metabolic pathways (Kyoto Encyclopedia of Genes and Genomes (KEGG)) were carried out using BLAST2GO v2.3.1 (http://www.blast2go.de)^56^ as previously described^55^. Homology searches were conducted remotely on the NCBI server by QBLAST using a sequential strategy. First, sequences were searched against the NCBI nr protein database using an *E*-value cutoff of 10^−3^, with predicted polypeptides of a minimum length of 15 amino acids. Second, sequences that did not retrieve BLASTx hits were searched again using BLASTn, against the NCBI nr nucleotide database, using an *E*-value cutoff of 10^−10^. EC codes and KEGG metabolic pathway annotations were generated from the direct mapping of GO terms to their enzyme code equivalents. Finally, InterPro searches were carried out remotely using BLAST2GO via the InterProEBI web server. To identify a non-redundant set of candidate AMP and lysozyme genes expressed in *N. vespilloides*, we established a reference set of known or predicted insect-derived AMPs and lysozymes using published sequences and by searching in-house and NCBI databases^19^. *Yarrowia* transcripts assembled *de novo* from the pooled carcass samples were functionally analysed using the KEGG automatic annotation server KAAS^57^ to assign KEGG Orthology (KO) identifiers and to map *Yarrowia*-like transcripts to KEGG pathways.

### Differential gene expression and GO enrichment analysis

Digital gene expression analysis was carried out using CLC Genomics Workbench v8.1 to generate BAM (mapping) files, and QSeq Software (DNAStar Inc., Madison, WI, USA) was then used to remap the Illumina reads from all five samples onto the reference transcriptome followed by counting the sequences to estimate expression levels, using previously described parameters for read mapping and normalization^55^. Biases in the sequence data sets and different transcript sizes were corrected using the RPKM algorithm to obtain correct estimates for relative expression levels. To control for the effect of global normalization using the RPKM method, we also analysed a number of highly conserved housekeeping genes, including several genes encoding ribosomal proteins (Rpl3, Rpl5, Rpl7, Rps4e, Rps5, Rps8, Rps18 and Rps24), NADH-dh, elongation factor-1α and eukaryotic translation initiation factors 4 and 5. The overall variation in expression levels across samples and treatments for these housekeeping genes was <1.3-fold between samples (based on log2-transformed RPKM values), indicating they were not differentially expressed. Because RNA-Seq data are noisier at lower expression levels, we excluded genes for which log2 RPKM <1 under any of our experimental conditions. RPL7 and RPS4e were used as reference genes and are shown in the heat map (Fig. 2) to verify the equivalent expression of control genes across tissues. Fisher's exact test was used as part of BLAST2GO to identify the overrepresentation of GO terms among lists of differentially expressed genes (eightfold change or higher) in the different gut section data sets relative to the complete reference data set that contained the assembly of all sequences. The GO-enriched bar charts were simplified to display only the most specific GO terms by removing parent terms representing existing child terms using the function ‘Reduce to most specific terms' in BLAST2GO. A GO term was considered significantly enriched if the *P* value corrected by false discovery rate control was <0.01.

### Specimens for profiling the microbiota

*N. vespilloides* larvae and pupae were the first generation derived from adults collected from three different wild populations in Germany in close proximity to Giessen (Albach, Schiffenberger Wald, Lamsbach and Annerod). The original adults that were collected are as follows: three females and four males from Schiffenberger Wald, three females and two males from Annerod and three females and males each from Lamsbach. From these we set up two mating pairs for each of the original populations. Six larvae (one from each breeding pair) were rinsed sequentially in distilled water, 1% SDS and sterile Millipore water and were then stored at −20 °C for DNA extraction. Pupae were allowed to complete metamorphosis, and emerging adults were maintained for several days in small plastic boxes with moist soil without access to food. Eight live beetles (four females and four males, reproductively mature and breeding) were frozen at –20 °C for 30 min and then surface sterilized with distilled water, 1% SDS and sterile Millipore water. The whole gut was dissected under sterile water and separated into two parts for DNA extraction: (1) the foregut and midgut, including the front part of the hindgut with a circular cross-section, and (2) the long, flat part of the hindgut (Fig. 1a). Anal secretions were collected from breeding adult beetles (different from those used for profiling the gut microbiota), pooled and stored at −20 °C for DNA extraction.

### Bacterial community profiling

DNA was extracted from adult gut sections, whole larvae and pooled anal secretions using the SoilMaster DNA Extraction Kit (Epicentre, Madison, WI, USA) according to the manufacturer's instructions, including lysozyme treatment to lyse Gram-positive cells. Corresponding aliquots of extracted DNA representing each life stage, sex and gut region were pooled and sequenced by bacterial TEFAP using the Gray28F and Gray519R 16*S* rRNA primers^58^. A sequencing library was generated by one-step PCR (30 cycles) using a mixture of HotStar and HotStar HiFidelity *Taq* polymerases (Qiagen, Hilden, Germany). Sequencing was extended from Gray28F using a Roche 454 FLX instrument with Titanium reagents and procedures at the Research and Testing Laboratory (Lubbock, TX, USA, http://www.medicalbiofilm.org/).

BTEFAP reads were analysed using QIIME^59^. Low-quality reads (quality cutoff=25) and sequences <200 bp were removed, resulting in 51,416 sequences for analysis (mean±s.d.=8,569±2,499 per sample). Sequencing noise was filtered using the denoise_wrapper implemented in QIIME^60^. Sequences were clustered into OTUs at 97% sequence identity using CD-HIT^61^ and UCLUST^62^ with a multiple OTU picking strategy. For each OTU, the most abundant sequence was chosen as a representative sequence, and taxonomy assignment was carried out with RDP classifier^63^ using the SILVA database^64^. An OTU table was generated in QIIME (Supplementary Table 1). Rarefaction curves were plotted by subsampling the OTU table with step increments of 100 sequences and 100 iterations at each step (Supplementary Fig. 10). A genus-level heat map was generated using the MultiExperiment Viewer^65^. Clustering of samples in QIIME was based on an unweighted UniFrac distance matrix^66^, and a phylogenetic tree of the representative sequences was reconstructed in FastTree (GTR model)^67^ after aligning the sequences using the SINA aligner^68^.

### Fungal community profiling

Part of the fungal LSU rRNA gene was amplified from six adult beetles (male midgut and hindgut, and female midgut and hindgut) and from anal secretions (collected from two adult beetles), using primers LS1 and LR5 as previously described^69^,^70^. Amplicons were cloned in *Escherichia coli* using the StrataClone PCR Cloning Kit (Agilent Technologies) according to the manufacturer's instructions. Inserts of 7–20 clones per sample were amplified using the flanking primers M13fwd and M13rev and sequenced unidirectionally with M13fwd. All sequences were compared with the NCBI nr database for taxonomy assignment. Representative sequences were aligned using SINA and imported into ARB^71^. Closely related fungal reference sequences were selected from the LSU reference database, and the alignment was exported into FastTree for phylogenetic reconstruction using the GTR model. The same beetle samples were also sequenced by 454 amplicon sequencing using the fungal ITS primers ITS1 and ITS4 (Research and Testing Laboratory). QIIME was used for quality trimming and analysis of the raw reads as described above for bacterial 16*S* sequences to generate an OTU table and to extract representative sequences for each OTU. Representative OTU sequences were compared against the NCBI nr database with BLAST2GO for taxonomy assignment.

### Diagnostic PCRs

Diagnostic primer pairs (Supplementary Table 4) were designed for *Morganella*, *Providencia*, *Proteus*, Neisseriaceae, Clostridiales, *Vagococcus*, Xanthomonadaceae and *Yarrowia* based on the aligned representative OTU sequences. The quality of the primers was assessed using Primer3 (ref. 72). Specificity was confirmed *in silico* by comparison with the RDP database, and *in vitro* by direct sequencing of amplicons obtained from beetle gut samples following the optimization of PCR conditions by gradient PCR. Clean sequences (no double signals) identical to the target sequences were taken as confirmation of primer specificity. The Enterobacteriaceae primer pair was obtained from the literature^73^. Diagnostic PCRs were prepared for all primer pairs with the individual DNA extracts of the six larval and eight adult beetles (midguts and hindguts, respectively). The PCR conditions are summarized in Supplementary Table 5.

#### *Yarrowia* qPCR

Quantitative real-time PCRs were carried out using a Bio-Rad CFXConnect instrument and a 20 μl reaction volume containing Absolute Blue QPCR SYBR (Thermo Scientific, Germany) according to the manufacturer's instructions. DNA extracts from pure cultures of two *Yarrowia* strains (strains B02 and C11 isolated from *N. vespilloides* anal secretions) were amplified using specific qPCR primers (fwd: 5′-TCAACAACGGATCTCTTGGC-3′ and rev: 5′-ATACCATACCGCGCAATGTG-3′) that target a 109 bp region of the ITS of *N. vespilloides*-associated *Yarrowia* strains. The products were purified from an agarose gel (Zymoclean gel DNA recovery kit, Zymo Research) and quantified using a Nanodrop spectrophotometer (PeqLab Biotechnology) before they were used to construct a standard curve of Ct values against DNA concentrations. Amplified DNA products were serially diluted from 10^−1^ to 10^−7^ ng μl^−1^ allowing the estimation of *Yarrowia* ITS copy numbers in unknown samples. For all three assays, the copy numbers of 28S and ITS genes were estimated from the regression of log DNA concentrations based on standards plotted against Ct values. For quantitative PCR (qPCR) analysis, DNA samples were diluted to a concentration of 1 ng μl^−1^, and 1 μl was used as a template for qPCR. Estimated DNA concentrations were corrected by the appropriate dilution factors, such that the final units of quantification were the number of gene copies per sample. The following qPCR cycler conditions were used: 95 °C for 15 min, 40 cycles of 95 °C for 15 s, 66 °C for 30 s and 72 °C for 20 s. The qPCR results were analysed using CFX Manager v3.1 (Bio-Rad) with an automatic cutoff for considering sample amplification as positive. All runs were followed by melt curve analysis that included raising the temperature of the reaction mixtures from *T*_a_ up to 95 °C (held for 10 s).

#### Diagnostic PCR

We used diagnostic PCR primers Yarrow -28S-fw d2 and Yarrow -28S-rev4 that are specific to the *Yarrowia* 28S rDNA isolated from the *N. vespilloides* hindgut and yield a 350 bp product^14^. The following PCR cycler conditions were used: 94 °C for 3 min, 38 cycles of 94 °C for 40 s, 65 °C for 40 s, 72 °C for 40 s and a final step at 72 °C for 4 min. We used 1 μl of template DNA and the products were visualized on 2% agarose gels run in TAE buffer.

#### Absence of *Yarrowia* in fresh cadavers

To exclude the presence of *Yarrowia* as part of the microbiome of fresh mouse cadavers before contact with the beetles, we sampled carrion DNA using two approaches. In the first approach (*n*=6), we extracted DNA from the external cadaver surface (hair and skin) using sterile DNA-free swabs. In the second approach (*n*=4), we extracted DNA from dissected guts, because the feeding cavity of the cadaver consists mostly of internal organs including the gut. DNA was extracted using a PowerSoil DNA isolation kit (MO BIO Laboratories, USA) according to the manufacturer's instructions. We used 1 μl of DNA as the template for diagnostic PCRs with the *Yarrowia*-specific 28S primers described above.

### Fluorescence *in situ* hybridization

Bacterial symbionts were localized in the gut of adult beetles by designing specific diagnostic fluorescence *in situ* hybridization probes for each taxon (Supplementary Table 6). Different regions of the guts of four adult beetles were dissected under sterile water, fixed in 70% ethanol and embedded in Technovit 8100 cold-polymerizing resin (Heraeus Kulzer, Hanau, Germany) as previously described^74^,^75^. Sections (10 μm) were prepared with a diamond knife on a Microm HM 355S microtome (Thermo Fisher Scientific), and hybridized at 50 °C with the specific Cy3-labelled probe for each taxon, combined with a Cy5-labelled general eubacterial probe (EUB338) and 4,6-diamidino-2-phenylindole. Fluorescence images were recorded with an AxioImager Z1 microscope (Carl Zeiss AG, Oberkochen, Germany) at 400 × magnification using the *z*-stack option to combine different focus planes and the mosaic tool.

### Isolation of yeast from anal secretions

The pooled anal secretions from five *N. vespilloides* females were diluted in two steps with 70% glycerol to obtain 1:5 and 1:50 dilutions. We spread 20 μl of each dilution on YPD agar (yeast extract, peptone, dextrose) containing 15 μg ml^−1^ tetracycline, 15 μg ml^−1^ nalidixic acid and 25 μg ml^−1^ chloramphenicol, and the plates were incubated at 25 °C for 24 h. Individual colonies were cultured in YPD broth, and DNA was extracted by lysing the pelleted cells in 0.2% SDS at 90 °C for 4 min, followed by centrifugation at 2,415 *g* for 4 min at room temperature. A fragment of the 28S rRNA gene was amplified from 0.5 μl DNA extract using the LS1/LR5 primer pair^69^,^70^ in a touchdown PCR procedure (94 °C for 2 min, 13 cycles of 94 °C for 1 min, 68 °C for 1 min (−0.7 °C/cycle), 72 °C for 1 min, and 26 cycles of 94 °C for 1 min, 58 °C for 1 min, 72 °C for 1 min and a final step of 72 °C for 6 min) resulting in an ∼900 bp amplicon. PCR products were purified using the Exo/FastAP protocol^76^ and sequenced from each end using the LS1 and LR5 primers, respectively. All sequences were compared with the NCBI nr database for taxonomy assignment. Sequences were aligned using MAFFT^77^ and maximum likelihood phylogenetic trees using 1,000 bootstrap replications were generated in MEGA6 (ref. 78).

### Analysis of extracellular metabolites by GC–MS

Two strains of *Yarrowia* spp. (B02 and C11) isolated from *N. vespilloides* secretions (as described above) were grown in liquid and solid media to investigate the production of extracellular metabolites. The liquid medium comprised 0.17% yeast nitrogen base, 2% glucose and 0.5% ammonium sulfate. The medium was inoculated with 4 × 10^4^ yeast cells and incubated at 27 °C for 24 h, before samples containing 1 × 10^6^ cells were collected from the suspension, quenched with an equal volume of cold (–80 °C) 100% methanol and incubated with a 100 μm solid-phase microextraction fibre coated with polydimethylsiloxane (Supelco, Sigma-Aldrich, St Louis, MO, USA) for 30 min. In addition, we used solid medium to resemble the natural growth of *Yarrowia* spp. on a meat substrate. A portion of ultraviolet-sterilized chicken meat (∼1 cm × 0.5 cm) was inoculated with 8 × 10^6^ B02 and C11 cells and incubated at 20 °C. After 2 weeks, the meat pieces were transferred to 200 μl cold (–80 °C) 100% methanol, vortexed at 2 °C for 3 min, centrifuged at 13,000 *g* for 2 min at 2 °C and 100 μl of the supernatant was collected and stored at –80 °C before GC–MS analysis of 1 μl samples. We used two different nutrient media to investigate metabolite production under simple conditions (glucose-containing medium) and more complex conditions (meat-containing medium) to cover a wider spectrum of growth conditions that may govern metabolite secretion. All samples from liquid and solid media were analysed using a Varian 450 GC coupled to a Varian 240MS ion-trap mass detector (Varian Inc., Palo Alto, CA, USA) equipped with a DB-5ms column (30 m × 0.25 mm diameter, film thickness 0.25 μm, Agilent Technologies). The temperature of the split/splitless GC injector was set to 250 °C and operated in splitless mode. The GC oven temperature was programmed as follows: 50 °C for 2 min, then heated at 10 °C per min to 300 °C and held for 5 min. Helium was used as a carrier gas at a constant flow rate of 1 ml min^−1^. External ionization spectra were recorded with a mass range of *m/z*=35–1,000. The ion source was kept at 160 °C and the ion trap at 90 °C, and the data were analysed using Varian MS Data Review v6.6. Compounds were identified by comparing mass spectra fragmentation profiles with the 2008 NIST mass spectral library. Control samples consisting of uninoculated solid and liquid media were also analysed, and compounds present in the controls were excluded.

### Data availability

Relevant sequence data, including RNA sequence data from mouse carcasses, and beetle bacterial and fungal community sequence data, have been deposited in the European Nucleotide Archive (ENA) with study accession number PRJEB18590 (http://www.ebi.ac.uk/ena/data/view/PRJEB18590). In particular, the carcass RNA-Seq data can be found with sample accession number ERS1473143, and the individual microbiome community profiling sequence sample files can be found with sample accession numbers ERS1473728 to ERS1473733. The cloned fungal 28S rDNA sequences have been deposited in the Genbank nucleotide database with accession codes KY657055 to KY657201. The authors declare that all other relevant data supporting the findings of this study are available within this article and its Supplementary Information, or from the corresponding authors on request.

## Additional information

**How to cite this article:** Vogel, H. *et al*. The digestive and defensive basis of carcass utilization by the burying beetle and its microbiota. *Nat. Commun.* **8,** 15186 doi: 10.1038/ncomms15186 (2017).

**Publisher's note:** Springer Nature remains neutral with regard to jurisdictional claims in published maps and institutional affiliations.

## Supplementary Material

Supplementary Information Supplementary Tables, Supplementary Figures and Supplementary References

Supplementary Data 1 Bacterial OTUs (taxonomic classification and representative sequences) associated with adult and larval *N. vespilloides* and anal secretions as revealed by TEFAP. Taxonomy was assigned to representative sequences using the RDP classifier implemented in QIIME.

## Figures and Tables

**Table 1:**
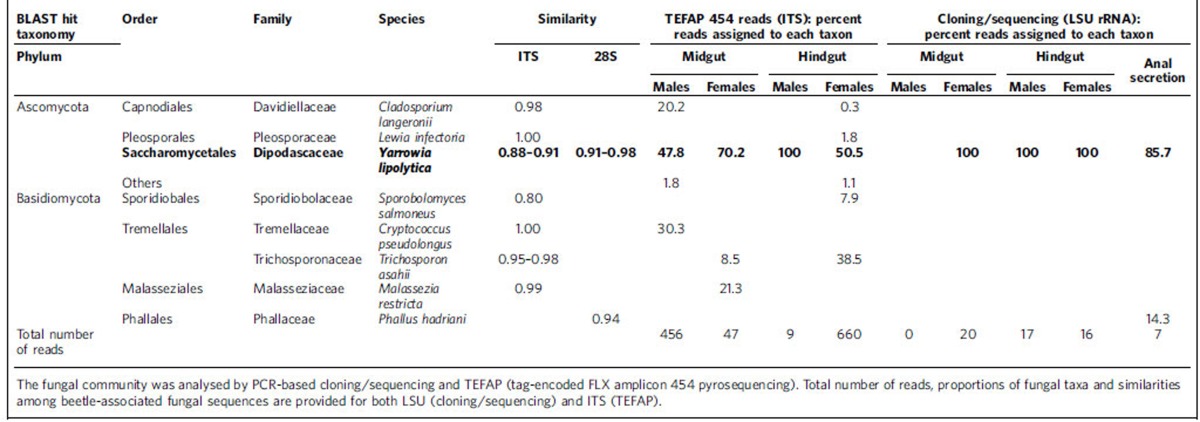
Fungal community in adult *N. vespilloides* and their anal secretions.
