# Supplementary material for: The digestive and defensive basis of carcass utilization by the burying beetle and its microbiota
Source: Nat Commun. 2017 May 9;8:15186. doi: 10.1038/ncomms15186 (PMC5436106; doi:10.1038/ncomms15186)
Supplement: Supplementary Information — Supplementary Tables, Supplementary Figures and Supplementary References [file ncomms15186-s1.pdf]

	Fat body	Foregut	Midgut	Hindgut	Rectum
Shannon index (H')	1.93	1.60	2.39	2.39	2.42

Supplementary Table 1: Diversity of AMPs and lysozymes expressed in different gut compartments. The Shannon diversity index is shown as H' = - Σ p_i (ln p_i), where p_i is the proportion of an ith type of AMP/lysozyme, for the 27 AMPs and 13 lysozymes expressed in the fat body, foregut, midgut and hindgut of *N. vespilloides* adults. The rectum showed a marginally higher Shannon index followed by the midgut and hindgut, with the foregut having the lowest value.

OTU ID	Midgut		Hindgut		Blast2Go hit	Similarity
	Males	Females	Males	Females		
10	1	0	0	0	*Yarrowia lipolytica*	0.91
27	0	0	0	1	*Trichosporon asahii*	0.95
31	17	1	0	37	*Candida galli/Yarrrowia lipolytica*	0.91/0.89
32	0	0	0	2	*Candida galli/Yarrrowia lipolytica*	0.91/0.89
34	0	0	0	1	*Trichosporon asahii*	0.97
57	176	32	6	278	*Yarrowia lipolytica*	0.89
75	0	0	0	12	*Lewia infectoria*	1.00
78	23	0	0	3	*Yarrowia lipolytica*	0.90
86	5	0	0	0	*Papulaspora* sp.	0.99
105	0	0	2	10	*Yarrowia lipolytica*	0.91
107	1	0	1	3	*Yarrowia lipolytica*	0.87
109	2	0	0	0	*Cladosporium langeronii*	0.97
111	0	0	0	1	*Trichosporon asahii*	0.97
131	3	0	0	0	*Lecanicillium psalliotae*	0.98
132	0	4	0	251	*Trichosporon asahii*	0.99
133	0	0	0	2	*Davidiella tassiana / Cladosporium cucumerinum*	0.99
134	90	0	0	2	*Cladosporium langeronii*	0.99
140	0	0	0	1	*Candida albicans*	0.97
157	0	0	0	52	*Sporobolomyces salmoneus*	0.8
162	0	0	0	4	*Debaryomyces hansenii*	0.99
165	138	0	0	0	*Cryptococcus pseudolongus*	1.00
167	0	10	0	0	*Malassezia restricta*	0.99
Total	456	47	9	660		

Supplementary Table 2: Fungal OTUs associated with adult *N. vespilloides* as characterized by TEFAP. Taxonomy was assigned to representative sequences using BLAST2GO. Sequences with best-matching hits against the host (*N. vespilloides* or other coleopteran sequences), its food (*Mus musculus*) or bacterial sequences, were discarded.

Kingdom	Phylum	Order	Family	Genus	Diagnostic PCR results					
					N. vespilloides adults			*N. vespilloides* larvae		
					positive	negative	percent positive	positive	negative	percent positive
Bacteria	Proteobacteria	Enterobacteriales	Enterobacteriaceae	general	8	0	100.0	6	0	100.0
			Enterobacteriaceae	*Morganella*	3	5	37.5	4	2	66.7
			Enterobacteriaceae	*Proteus*	8	5	100.0	6	0	100.0
			Enterobacteriaceae	*Providencia*	8	5	100.0	6	0	100.0
		Xanthomonadales	Xanthomonadaceae	*Ignatszchineria/ Wohlfahrtiimonas*	7	1	87.5	6	0	100.0
		Neisserales	Neisseria	*Neisseria*	5	3	62.5	6	0	100.0
	Firmicutes	Lactobacillales	Enterococcaceae	*Vagococcus*	7	1	87.5	6	0	100.0
		Clostridiales	Family Xi. Incertae Sedis	*Tissierella*	7	1	87.5	6	0	100.0
Fungi	Ascomycota	Saccharomycetales	Dipodascaceae	*Yarrowia*	7	1	87.5	6	0	100.0

Supplementary Table 3: Results of diagnostic PCRs for major bacterial and fungal taxa in *N. vespilloides* adult guts and larvae.

**Kingdom**	**Phylum**	**Order**	**Family**	**Genus**	**Primers**	**PCR cycle number**	**T_a (°C)**
Bacteria	Proteobacteria	Enterobacteriales	Enterobacteriaceae	general	En-Isu-3F/3R	40	60
	Proteobacteria	Enterobacteriales	Enterobacteriaceae	*Morganella*	Morgan-16S_fwd1/rev1	38	65
	Proteobacteria	Enterobacteriales	Enterobacteriaceae	*Proteus*	Proteus-16S-fwd1/rev1	38	67
	Proteobacteria	Enterobacteriales	Enterobacteriaceae	*Providencia*	Provid-16S-fwd1/rev1mod	32	67
	Proteobacteria	Xanthomonadales	Xanthomonadaceae	*Ignatzschineria/ Wohlfahrtiimonas*	Xantho-16S_fwd1/rev1	38	65
	Proteobacteria	Neisseriales	Neisseriaceae	*Neisseria*	Neiss_16S-fwd1/rev1	38	67
	Firmicutes	Lactobacillales	Enterococcaceae	*Vagococcus*	Vagoc-16S_fwd1/rev1	38	65
	Firmicutes	Clostridiales	Family XI. Incertae Sedis	*Tissierella*	Tissier-16S_fwd1/rev1	38	65
Fungi	Ascomycota	Saccharomycetales	Dipodascaceae	*Yarrowia*	Yarrow_26S-fwd2/rev4	38	66

Supplementary Table 4: PCR conditions used for diagnostic PCRs to confirm the presence of bacterial and fungal taxa in *N. vespilloides* adults and larvae.

	Probe name	Probe target	Probe sequence	Reference
1	EUB338-Cy5	General bacteria 16S rRNA	GCTGCCTCCCGTAGGAGT	Amann et al. 1990[1]
2	Cy3-Morgan_16S	*Morganella morganii* 16S rRNA	ATGGGTTCATCTGATGGTGC	This study
3	Cy3-Proteus_16S	*Proteus* 16S rRNA	CTCCTGTTACCGCTCGACTT	This study
4	Cy3-Provid_16S	*Providencia* 16S rRNA	GTCAGCAAGAAGCAAGCTTC	This study
5	Cy3-Xantho_16S	Xanthomonadales 16S rRNA	TCCCCTGCTTTAAACCGTAG	This study
6	Cy3-Tissier_16S	*Tissierella*-like 16S rRNA	ACAAGCTAATGGGACGCGAG	This study
7	Cy3-Vagoc_16s	*Vagococcus* 16S rRNA	GCTATGCATCACGGTCTTG	This study
8	Cy3-Neiss_16S	Neisseriales 16S rRNA	GTCGGCAGAAGAAGCAAGCT	This study
9	Cy3-Yarrow_28S	*Yarrowia lipolytica* 28S rRNA	TGAGTGTTAGGGTGRCAAAC	This study

Supplementary Table 5: Details of FISH probes used to localize general bacteria and major bacterial taxa in *N. vespilloides* gut regions identified by the TEFAP analysis of 16*S* amplicons.

(A) Carbohydrate metabolism pathways

Pathway	KEGG reference hierarchy (KO)
Glycolysis / Gluconeogenesis	ko00010
Citrate cycle (TCA cycle)	ko00020
Pentose phosphate pathway	ko00030
Pentose and glucuronate interconversions	ko00040
Fructose and mannose metabolism	ko00051
Galactose metabolism	ko00052
Ascorbate and aldarate metabolism	ko00053
Starch and sucrose metabolism	ko00500
Amino sugar and nucleotide sugar metabolism	ko00520
Inositol phosphate metabolism	ko00562
Pyruvate metabolism	ko00620
Glyoxylate and dicarboxylate metabolism	ko00630
Propanoate metabolism	ko00640
Butanoate metabolism	ko00650
C5-Branched dibasic acid metabolism	ko00660
Inositol phosphate metabolism	ko00562

(B) Lipid metabolism pathways

Pathway	KEGG reference hierarchy (KO)
alpha-Linolenic acid metabolism	ko00592
Arachidonic acid metabolism	ko00590
Biosynthesis of unsaturated fatty acids	ko01040
Ether lipid metabolism	ko00565
Fatty acid biosynthesis	ko00061
Fatty acid degradation	ko00071
Fatty acid elongation	ko00062
Glycerolipid metabolism	ko00561
Glycerophospholipid metabolism	ko00564
Secondary bile acid biosynthesis	ko00121
Sphingolipid metabolism	ko00600
Steroid biosynthesis	ko00100
Steroid hormone biosynthesis	ko00140
Synthesis and degradation of ketone bodies	ko00072

(C) Amino acid metabolism pathways

Pathway	KEGG reference hierarchy (KO)
Alanine, aspartate and glutamate metabolism	ko00250
Arginine and proline metabolism	ko00330
Arginine biosynthesis	ko00220
Cysteine and methionine metabolism	ko00270
Glycine, serine and threonine metabolism	ko00260
Histidine metabolism	ko00340
Lysine biosynthesis	ko00300
Lysine degradation	ko00310
Phenylalanine metabolism	ko00360
Phenylalanine, tyrosine and tryptophan biosynthesis	ko00400
Tryptophan metabolism	ko00380
Tyrosine metabolism	ko00350
Valine, leucine and isoleucine biosynthesis	ko00290
Valine, leucine and isoleucine degradation	ko00280

(D) Vitamin metabolism pathways

Pathway	KEGG reference hierarchy (KO)
Vitamin B6 metabolism	ko00750
Ubiquinone and other terpenoid-quinone biosynthesis	ko00130
Thiamine metabolism	ko00730
Riboflavin metabolism	ko00740
Retinol metabolism	ko00830
Porphyrin and chlorophyll metabolism	ko00860
Pantothenate and CoA biosynthesis	ko00770
Nicotinate and nicotinamide metabolism	ko00760
Folate biosynthesis	ko00790
Biotin metabolism	ko00780

Supplementary Table 6: Metabolic pathways detected in *Yarrowia* transcripts from carcass surfaces used by breeding *N. vespilloides* beetles. For all tables, only those pathways are listed for which at least three gene entries were detected.

	Function on carcass	Enzyme	E.C. number	Detected on carcass	KEGG ortholog	RPKM
1	Triglyceride digestion	Extracellular lipase	3.1.1.3	Yes	KO1046	1.74
				Yes	KO17900	6.02
				Yes	KO14675	1.99
				Yes	KO14674	1.37
2	Protein digestion	Extracellular protease	3.4.23	Yes		1.42
			3.4.23	Yes		1.11
			3.4.23	Yes		2.05
			3.4.21	Yes		8.98
			3.4.21	Yes		9.88
			3.4.21	Yes		3.61
			3.4.21	Yes		2.59
			3.4.21	Yes		1.48
			3.4.21	Yes		1.70
			3.4.22	No		
			3.4.17	Yes		1.91
			3.4.17	Yes		2.92
			Others	Yes		6.09
3	Creatine digestion	Creatinase	3.5.3.3	No		
			2.7.3.2	No		

Supplementary Table 7: Detection of *Yarrowia* transcripts representing extracellular lipases, proteases and creatinases, which could potentially facilitate the digestion of carcass lipids, proteins and creatine. Results from the KEGG-KAAS annotation pipeline were screened for the presence of any extracellular proteases and lipases, and those known to occur in the closely related *Y. lipolytica* [2, 3]. Creatinases were identified using pathways involving the breakdown of creatine associated with its KEGG entry (compound C00300).

A) High in Foregut vs. Midgut - Differential GO-term distribution

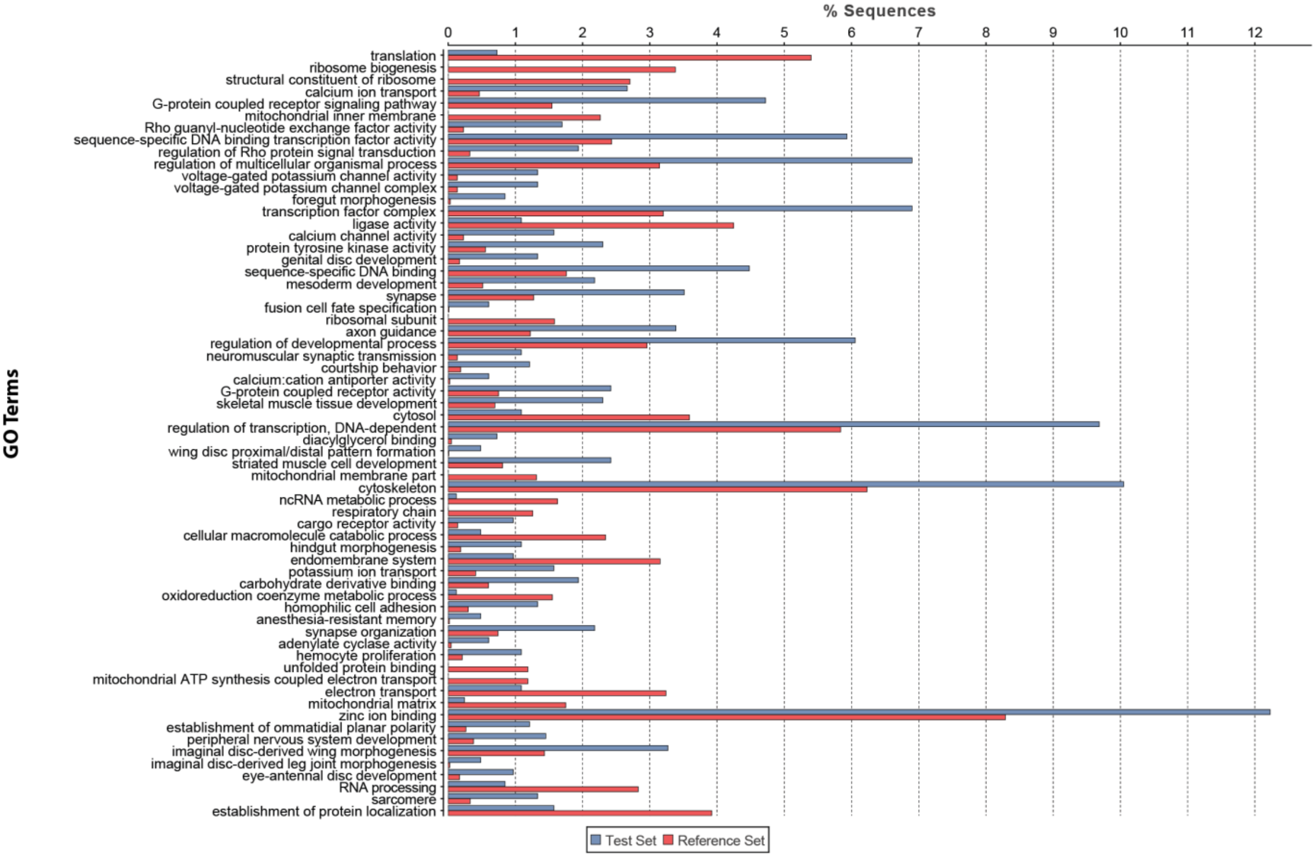

B) High in Midgut vs. Foregut - Differential GO-term distribution

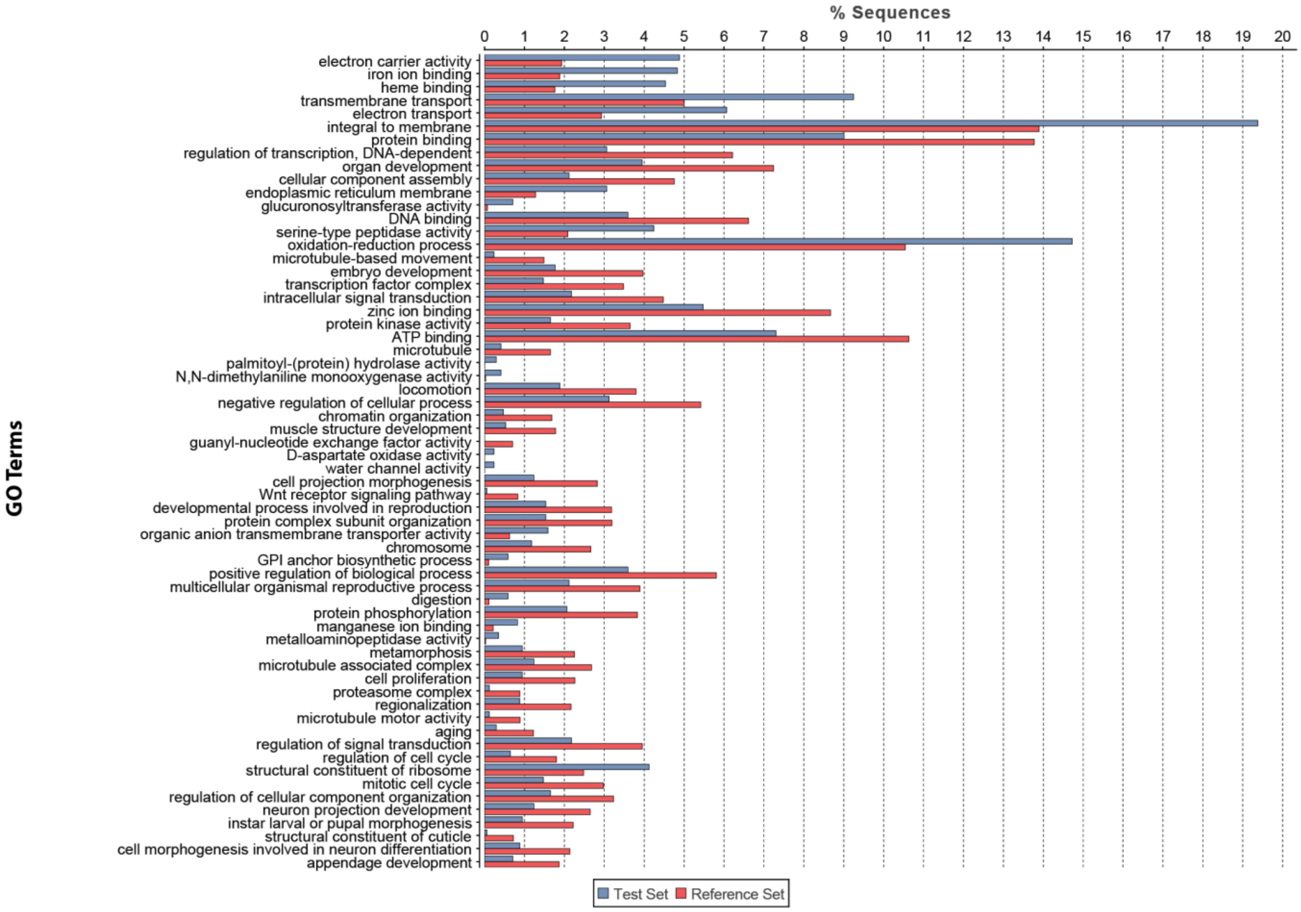

C) High in Midgut vs. Hindgut - Differential GO-term distribution

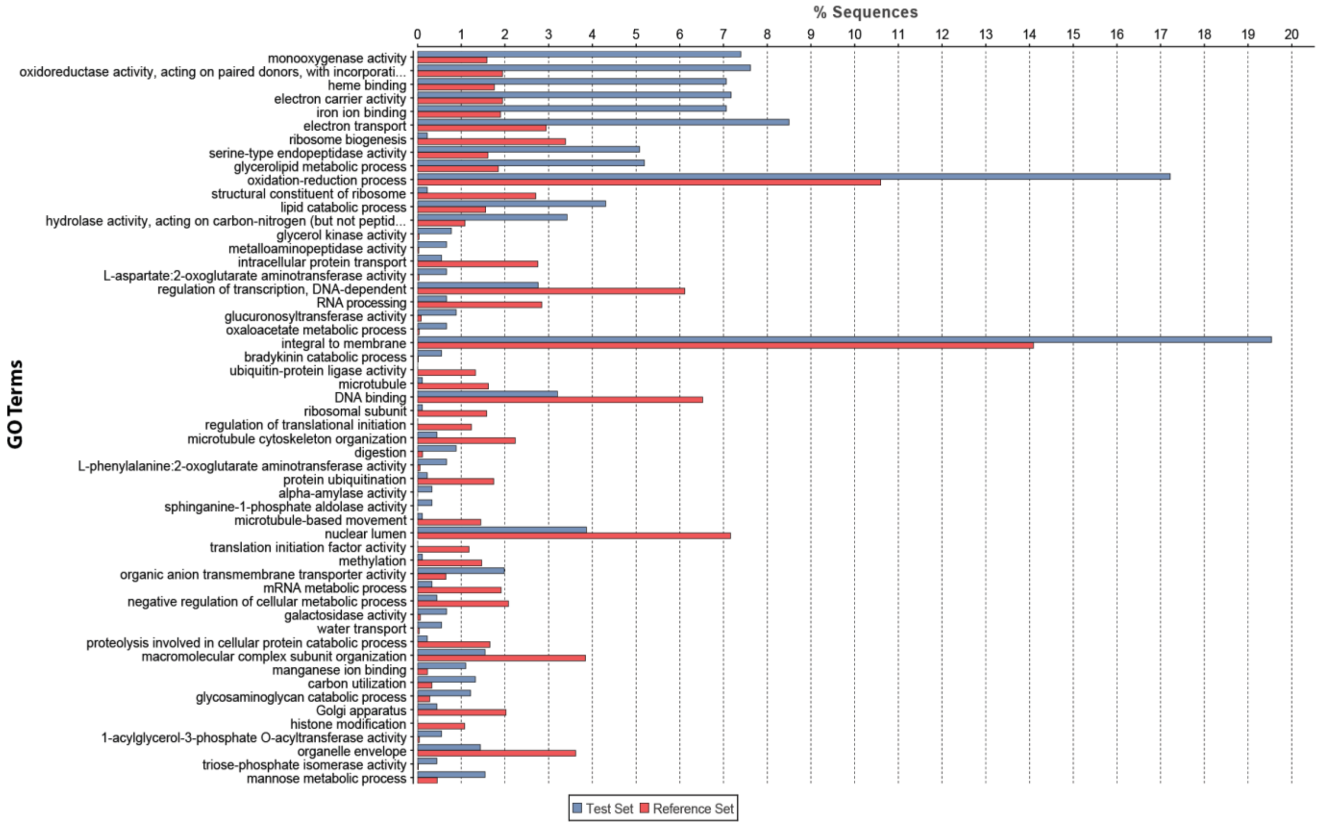

D) High in Hindgut vs. Midgut - Differential GO-term distribution

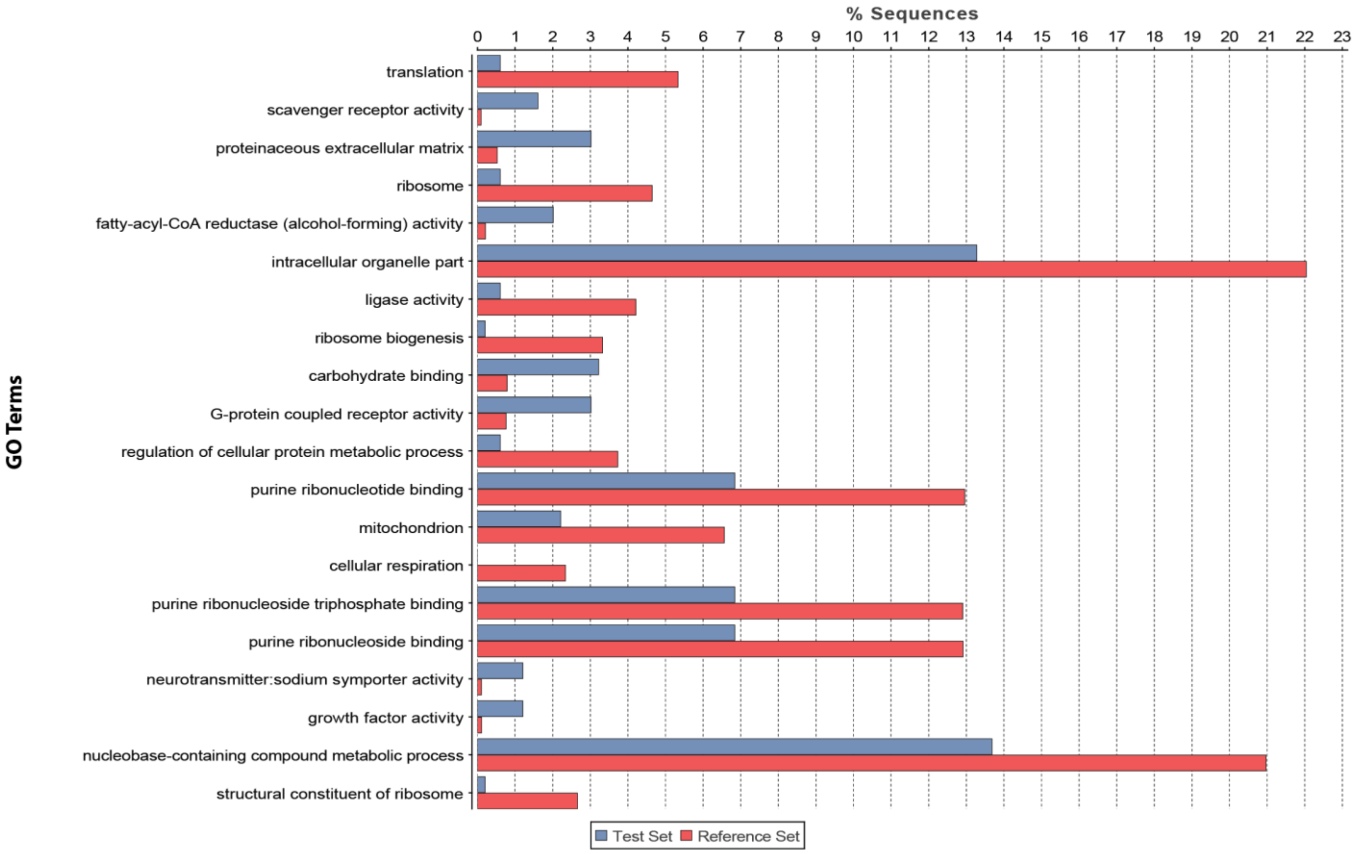

E) High in Hindgut vs. Rectum - Differential GO-term distribution

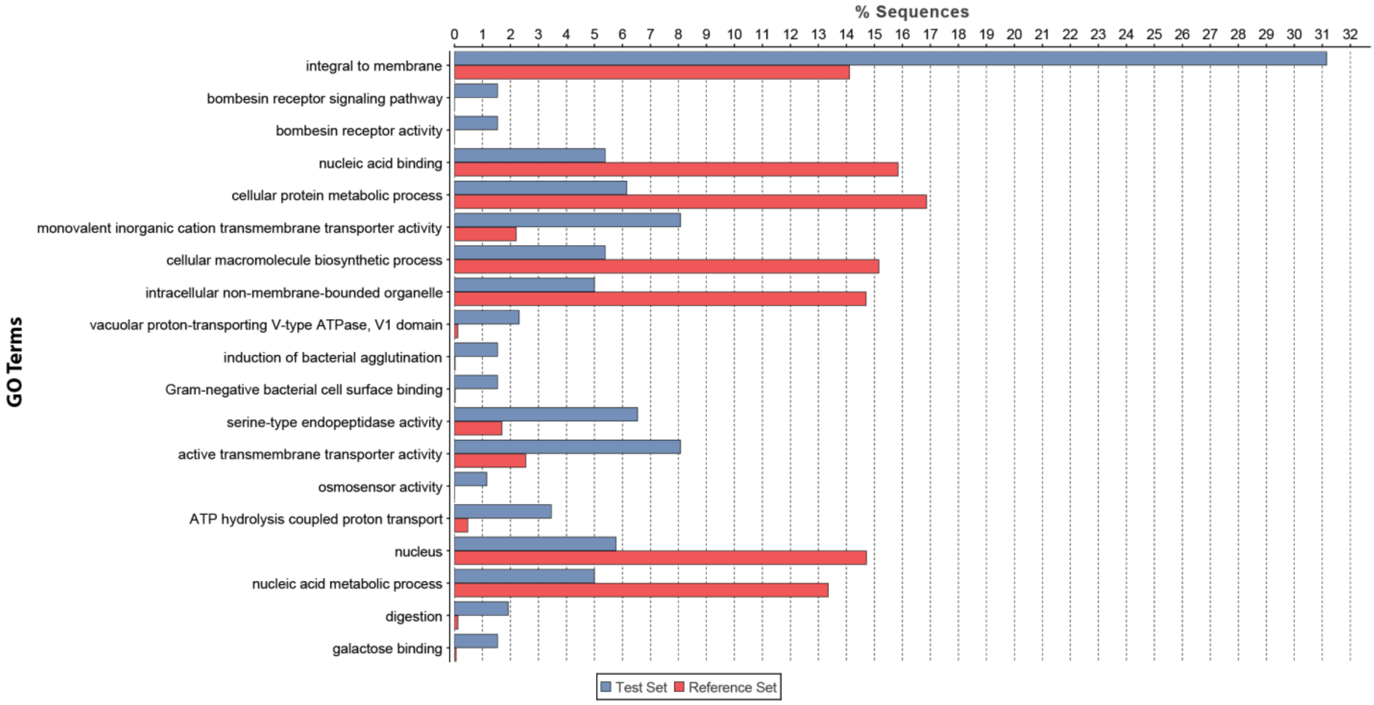

F) High in Rectum vs. Hindgut - Differential GO-term distribution

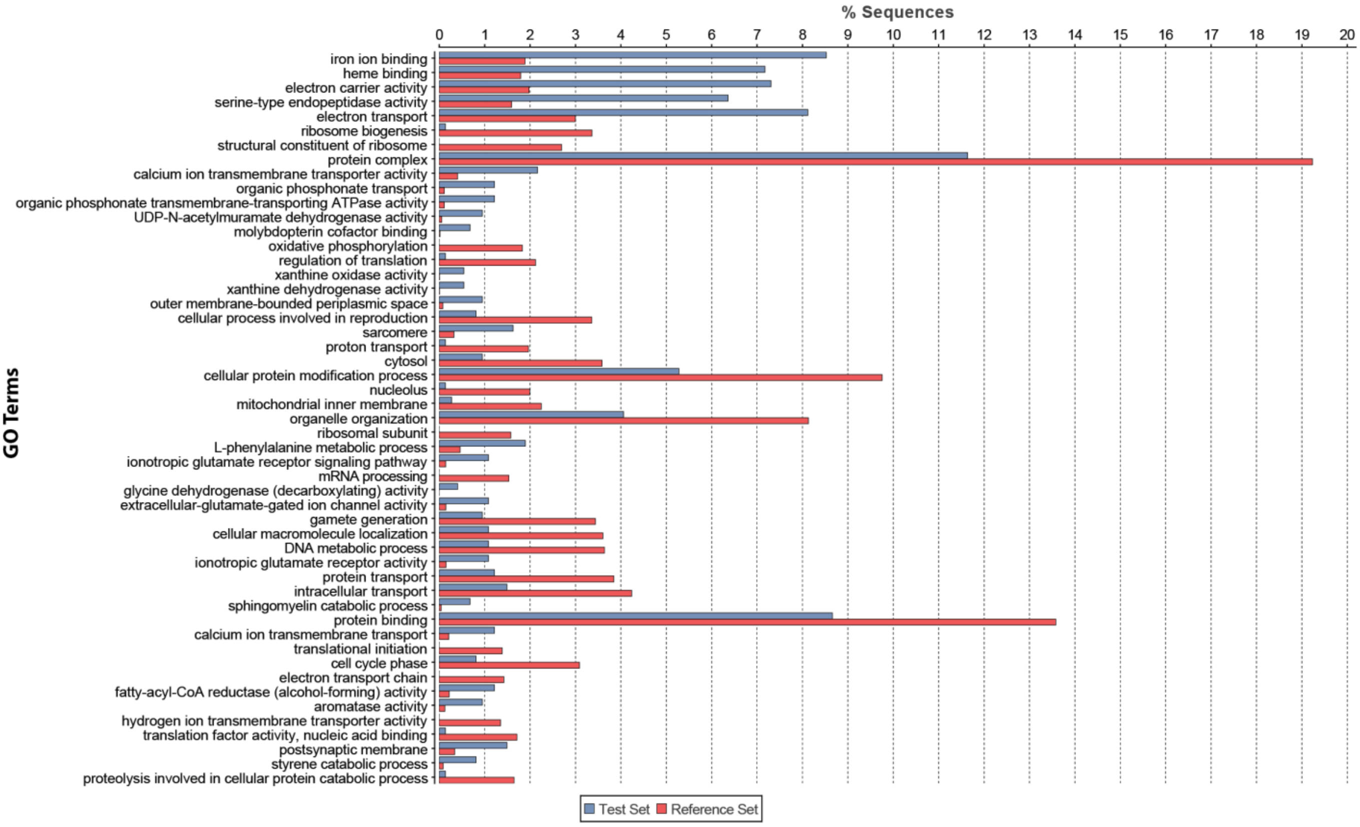

Supplementary Figure 1: Distribution of GO terms in different gut regions. Bar charts show the GO terms that were significantly (FDR < 0.01) enriched in the different gut sections of *N. vespilloides*. The GO terms are sorted in ascending order according to their FDR value, starting with the most significantly enriched. Only the most specific GO terms are displayed. Differences are shown as the percentage of sequences associated with a specific GO category in the test set (total number of differentially expressed contigs between different gut sections) versus the reference set (transcriptome backbone assembly) using Fisher’s exact test in BLAST2GO-PRO.

Neuropeptide & Hormone Signaling

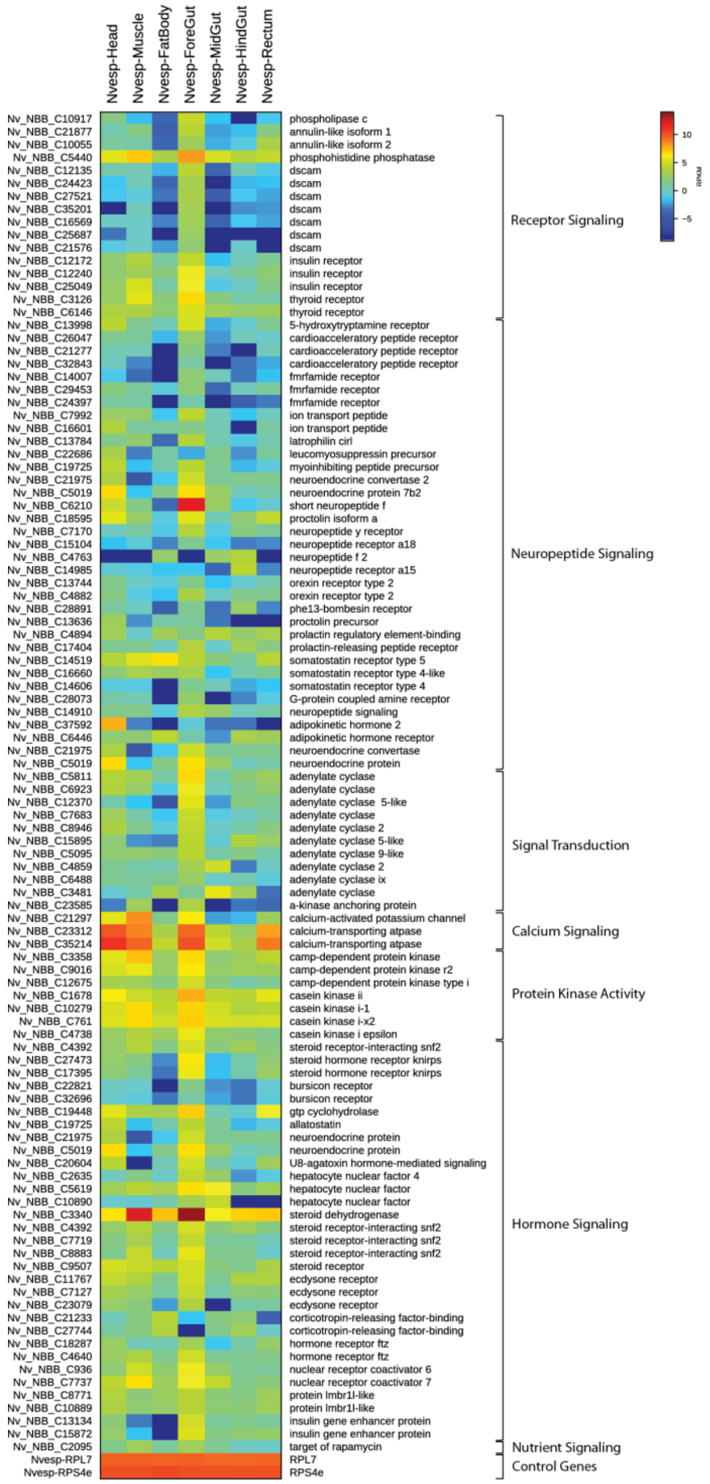

Supplementary Figure 2: Heat map showing the relative expression levels of neuropeptide and hormone signaling-related genes in different *N. vespilloides* tissues and gut sections. RPL7 and RPS4e are used as housekeeping genes and are shown to confirm the uniform expression of these control genes across tissues. The map is based on log2-transformed RPKM values (blue represents weakly-expressed genes, and red represents strongly-expressed genes).

Carbohydrate Degradation

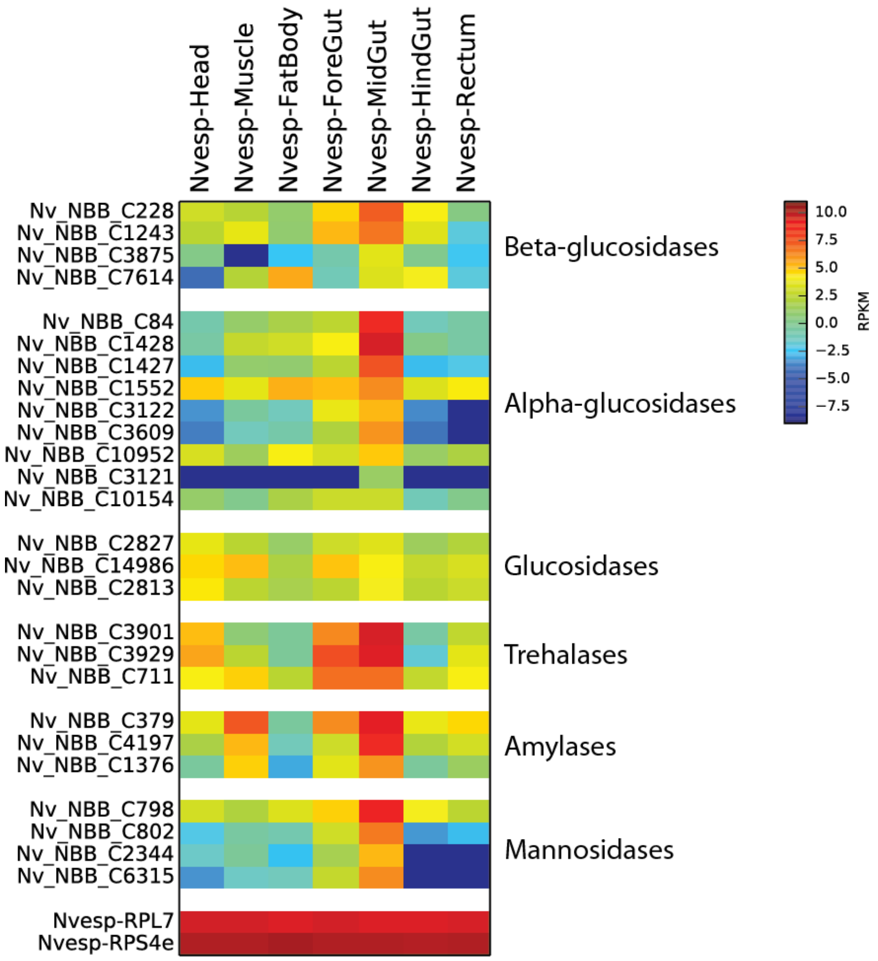

Supplementary Figure 3: Heat map showing the relative expression levels of carbohydrate degradation-related genes in different *N. vespilloides* tissues and gut sections. RPL7 and RPS4e are used as housekeeping genes and are shown to confirm the uniform expression of these control genes across tissues. The map is based on log2-transformed RPKM values (blue represents weakly-expressed genes, and red represents strongly-expressed genes).

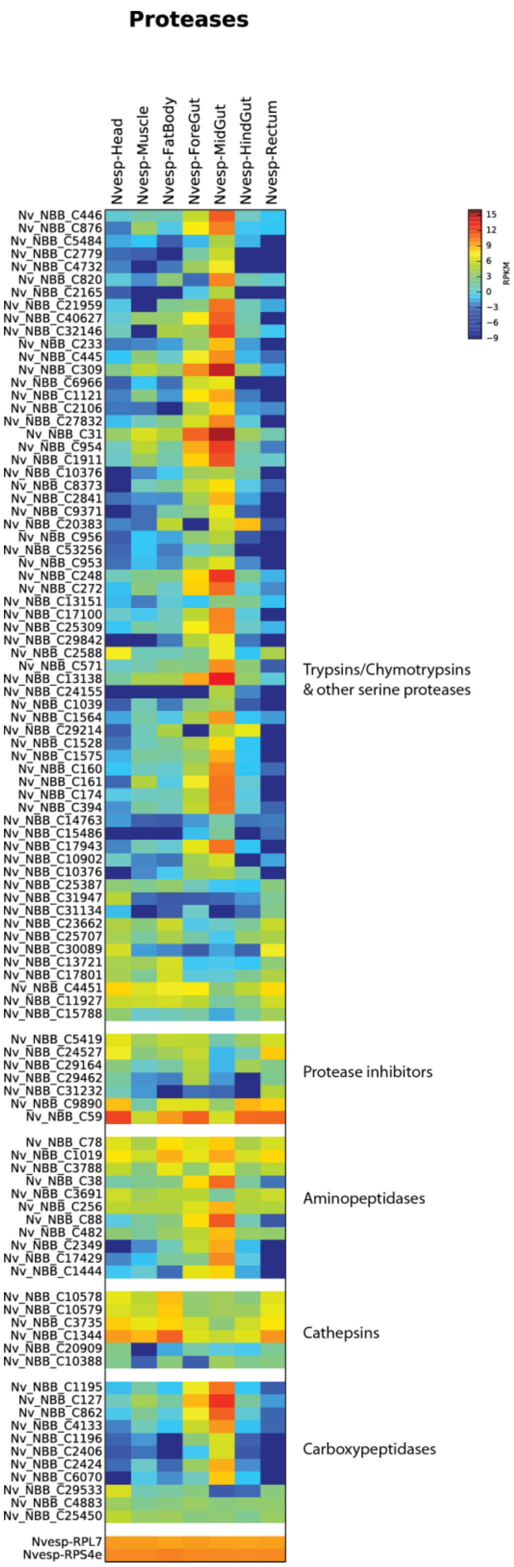

Supplementary Figure 4: Heat map showing the relative expression levels of protease genes in different *N. vespilloides* tissues and gut sections. RPL7 and RPS4e are used as housekeeping genes and are shown to confirm the uniform expression of these control genes across tissues. The map is based on log2-transformed RPKM values (blue represents weakly-expressed genes, and red represents strongly-expressed genes).

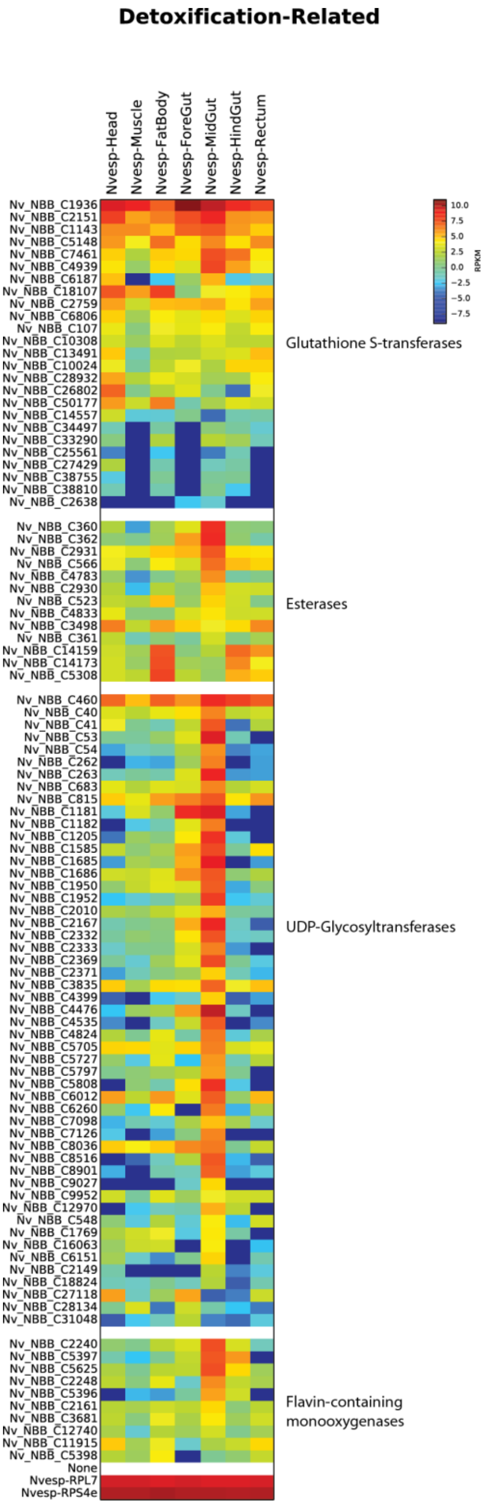

Supplementary Figure 5: Heat map showing the relative expression levels of putative detoxification-related genes in different *N. vespilloides* tissues and gut sections. RPL7 and RPS4e are used as housekeeping genes and are shown to confirm the uniform expression of these control genes across tissues. The map is based on log2-transformed RPKM values (blue represents weakly-expressed genes, and red represents strongly-expressed genes).

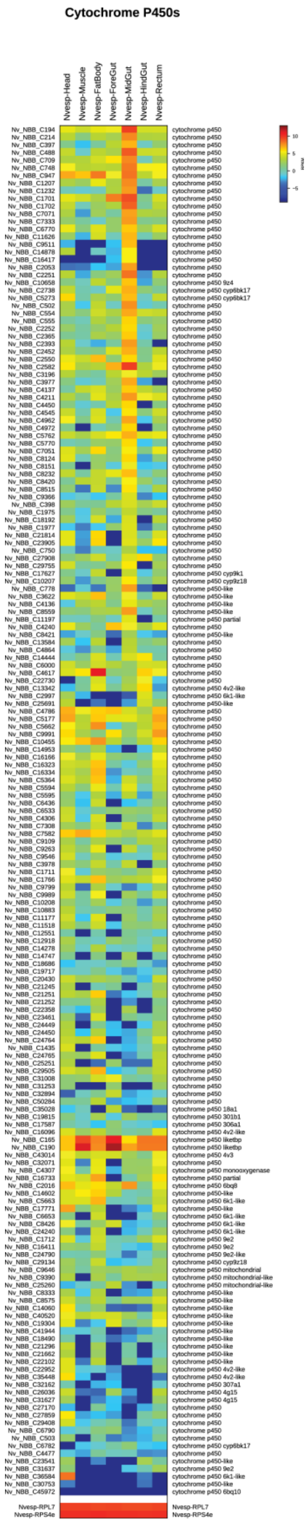

Supplementary Figure 6: Heat map showing the relative expression levels of cytochrome P450 genes in different *N. vespilloides* tissues and gut sections. RPL7 and RPS4e are used as housekeeping genes and are shown to confirm the uniform expression of these control genes across tissues. The map is based on log2-transformed RPKM values (blue represents weakly-expressed genes, and red represents strongly-expressed genes).

Transport, Binding, etc.

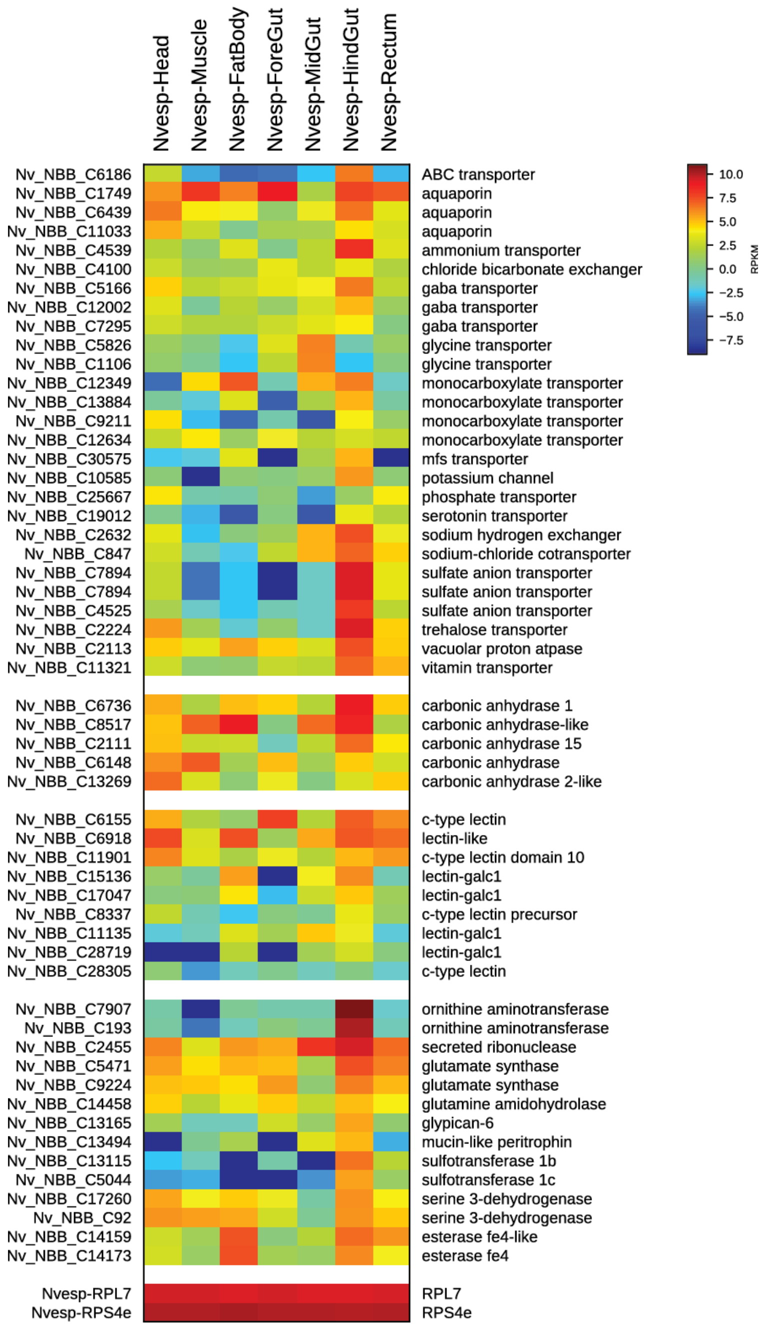

Supplementary Figure 7: Heat map showing the relative expression levels of genes related to transport and substrate binding processes in different *N. vespilloides* tissues and gut sections. RPL7 and RPS4e are used as housekeeping genes and are shown to confirm the uniform expression of these control genes across tissues. The map is based on log2-transformed RPKM values (blue represents weakly-expressed genes, and red represents strongly-expressed genes).

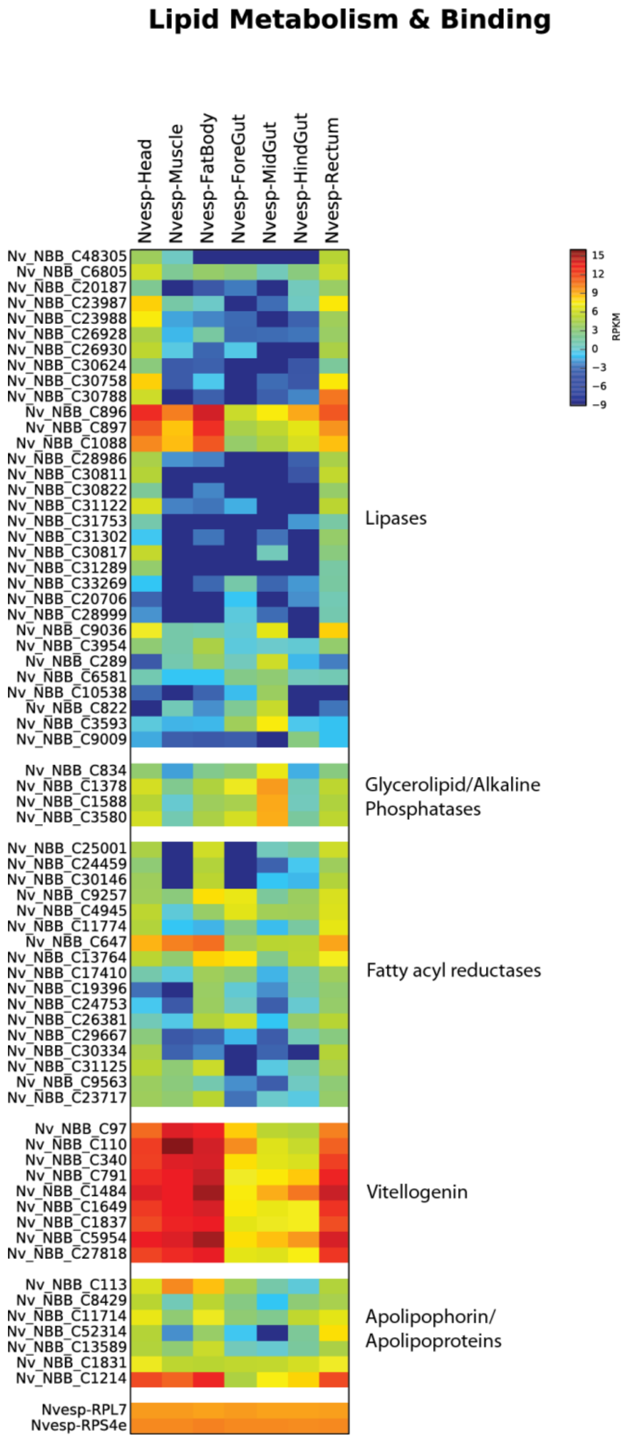

Supplementary Figure 8: Heat map showing the relative expression levels of genes related to lipid metabolism and lipid binding in different *N. vespilloides* tissues and gut sections. RPL7 and RPS4e are used as housekeeping genes and are shown to confirm the uniform expression of these control genes across tissues. The map is based on log2-transformed RPKM values (blue represents weakly-expressed genes, and red represents strongly-expressed genes).

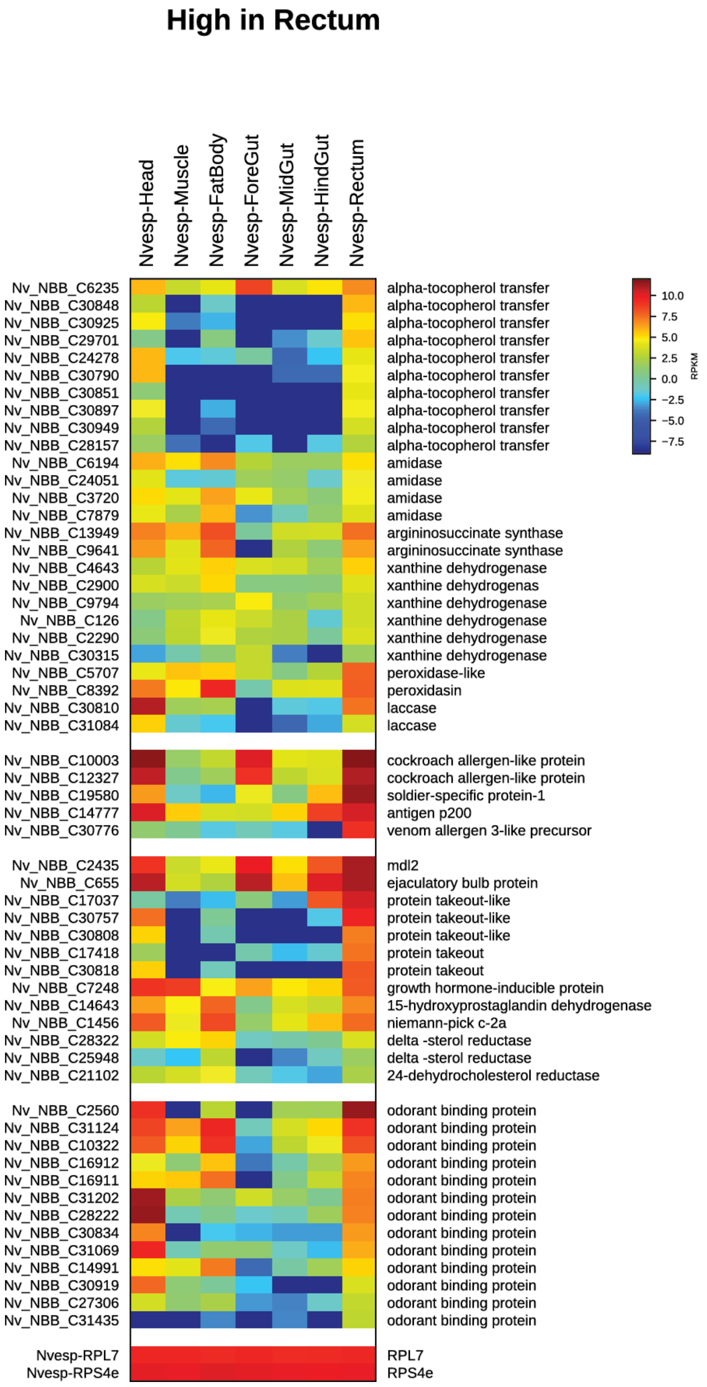

Supplementary Figure 9: Heat map of genes which show high expression levels in the rectum of *N. vespilloides*. RPL7 and RPS4e are used as housekeeping genes and are shown to confirm the uniform expression of these control genes across tissues. The map is based on log2-transformed RPKM values (blue represents weakly-expressed genes, and red represents strongly-expressed genes)

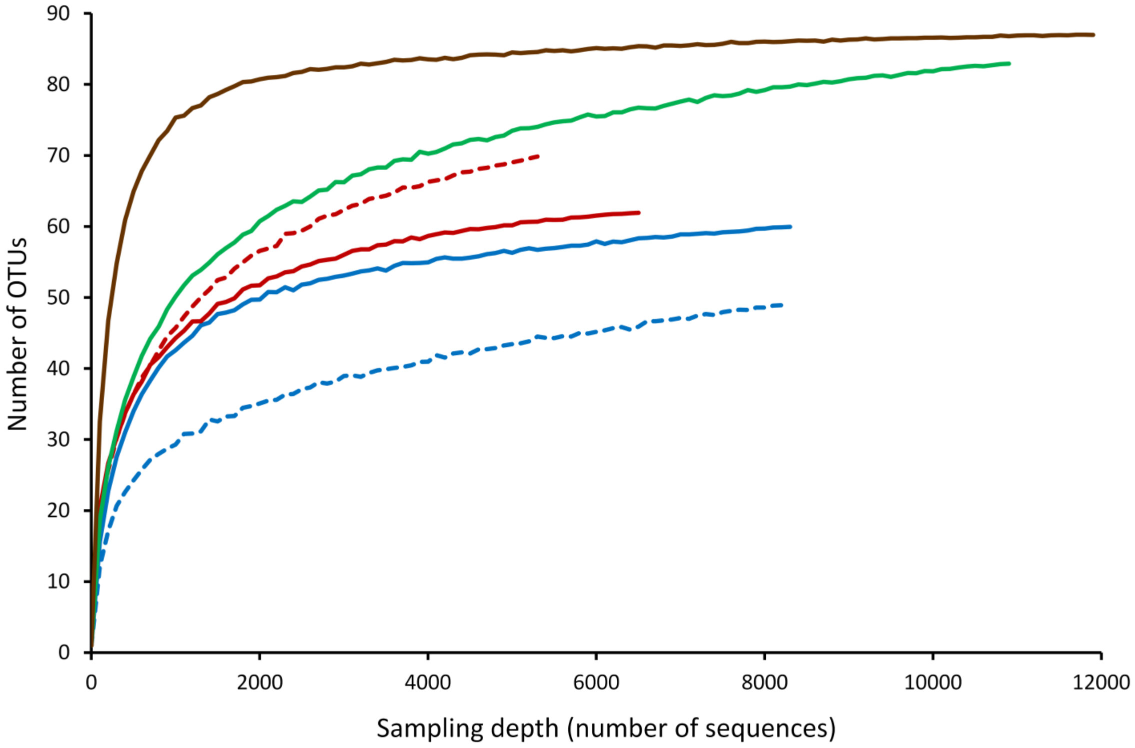

Supplementary Figure 10: Rarefaction curves showing TEFAP sequencing depth of bacterial 16*S* rRNA amplicons obtained from *N. vespilloides* adults and different life stages. Green = larvae, blue solid = female midgut, blue dashed = female hindgut, red solid = male midgut, red dashed = male hindgut, brown = anal secretion.

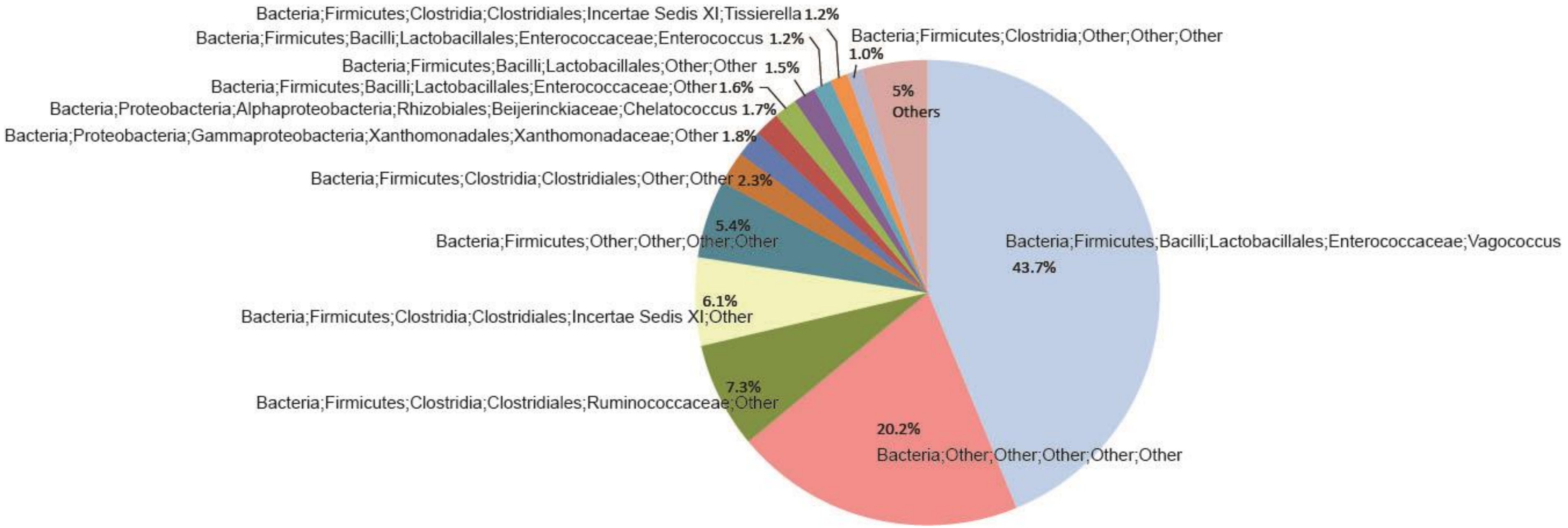

Supplementary Figure 11: Bacterial OTU composition of *N. vespilloides* anal secretions based on 16*S* rRNA gene amplicons sequenced by TEFAP. OTUs are collapsed at the genus level.

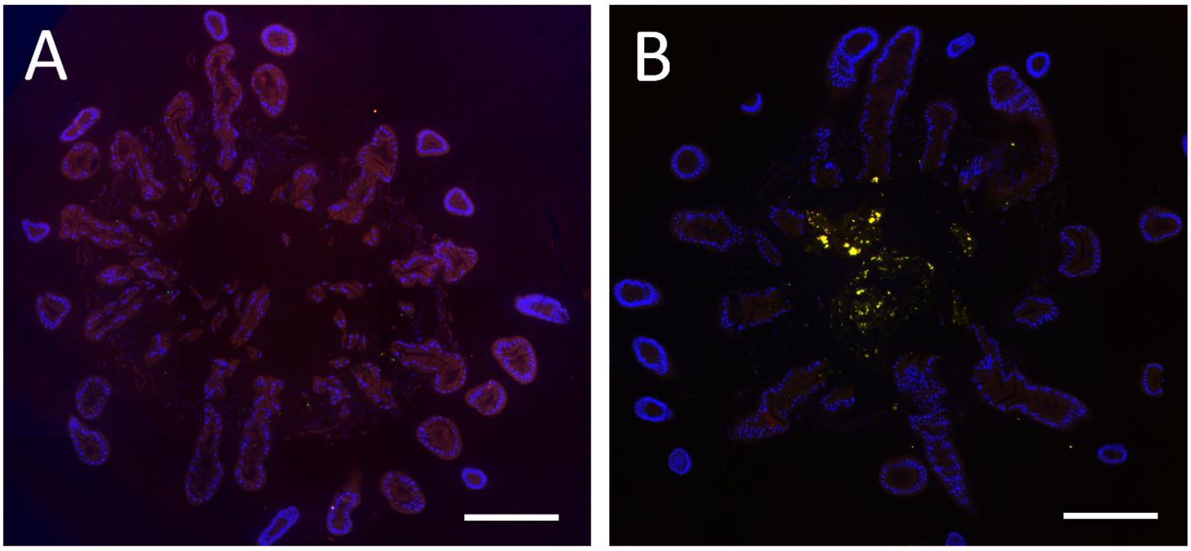

Supplementary Figure 12: Representative fluorescence micrograph of midgut sections from two female *N. vespilloides* beetles showing (A) low/undetectable prevalence of bacteria in some sections of the midgut, and (B) prevalence of *Vagococcus* but no other major bacterial taxa in the midgut. Bacteria were stained with the general bacterial probe EUB338-Cy5 (green) and a taxon-specific Cy3-labeled oligonucleotide probe (red). Nuclei were counterstained with DAPI (blue). Scale bars are 200 µm.

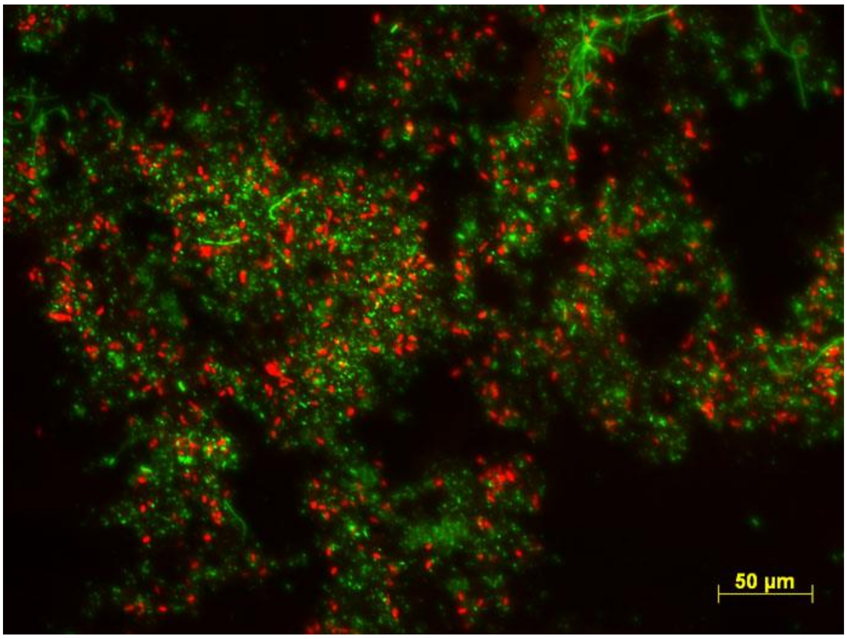

Supplementary Figure 13: Fluorescence micrograph of a suspension prepared from the rectum of a female *N. vespilloides* beetle showing the high abundance of the *Yarrowia*-related yeast species. Bacteria were stained with the eubacterial probe EUB338-Cy5 (green) and *Yarrowia* was stained with a specific Cy3-labeled oligonucleotide probe (r

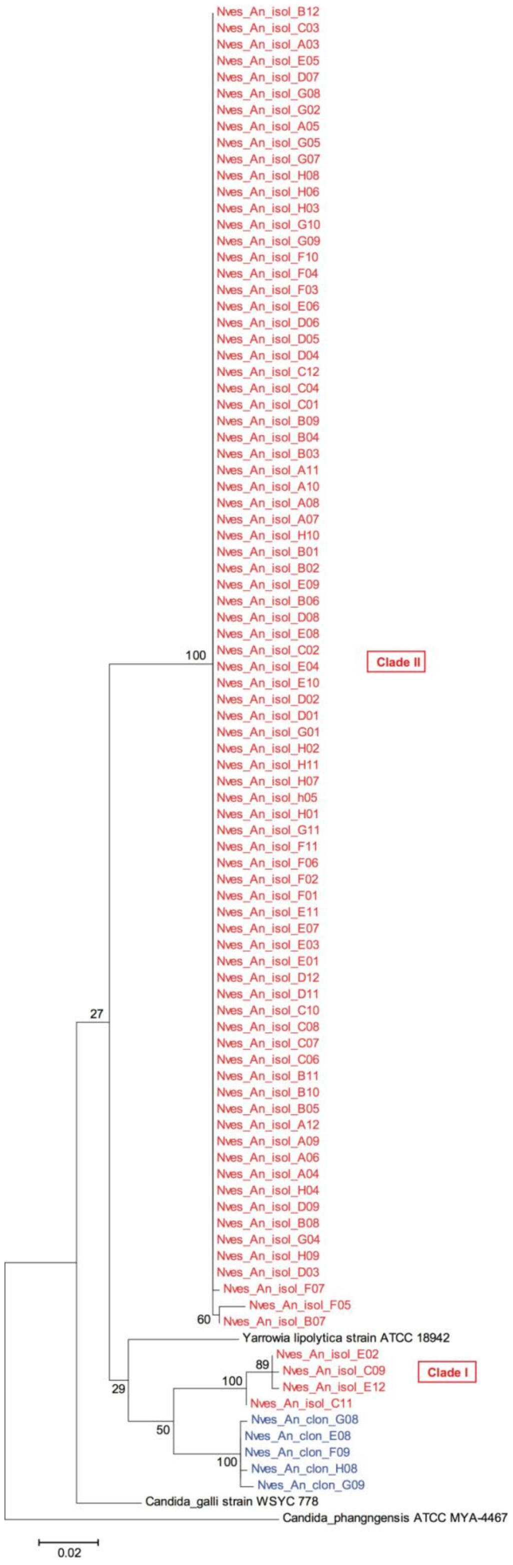

Supplementary Figure 14: Maximum likelihood phylogeny of the partial 28*S* rRNA gene sequences from 85 *Yarrowia* strains isolated from *N. vespilloides* anal secretions. The *Yarrowia* strains formed two distinct clades (clade I and II, represented in red), both closely related to *Y. lipolytica,* and those obtained from cloning/sequencing (also from anal secretions, and represented in blue). Numbers indicate bootstrap support values.

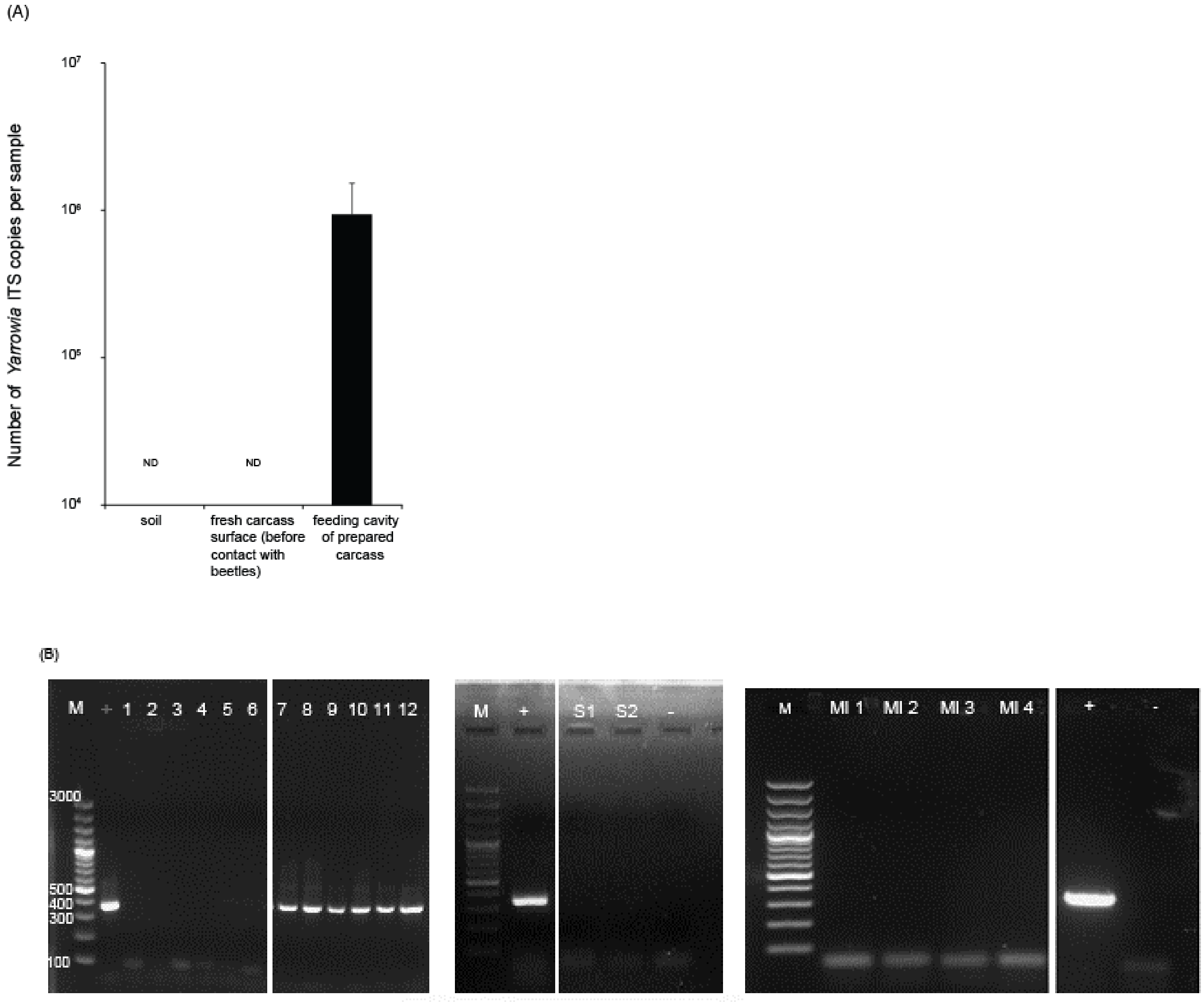

Supplementary Figure 15: Detection of *Yarrowia*-like yeasts using qPCR and diagnostic PCR: (A) qPCR analysis using *Yarrowia*-specific ITS primers: *Yarrowia* was not detected in the soil (n=2) or on the surface of fresh carcasses that had no contact with the soil or *N. vespilloides* beetles (n=6). However, *Yarrowia* was detected in the feeding cavity of carcasses prepared by the beetles (n=6). (B) Diagnostic PCR analysis using *Yarrowia*-specific 28S primers: *Yarrowia* DNA was not detected in the soil (n=2; labeled S1, S2 in the figure) that was used to rear the beetles, on the surface of fresh mouse cadavers (lanes 1–6) or inside fresh cadavers (DNA from cadaver guts, labeled MI1-MI4) but was present on carcasses used for breeding by *N. vespilloides* (lanes 7–12) as a 350-bp product comparable to the single band of the same length seen in pure *Yarrowia* genomic DNA (labeled '+' indicated by red arrow). *Yarrowia* was most likely introduced onto the carcass by the beetles through anal secretions (see main text). ND = not detected; M = DNA marker (3000 to 100 bp); – = blank PCR control.

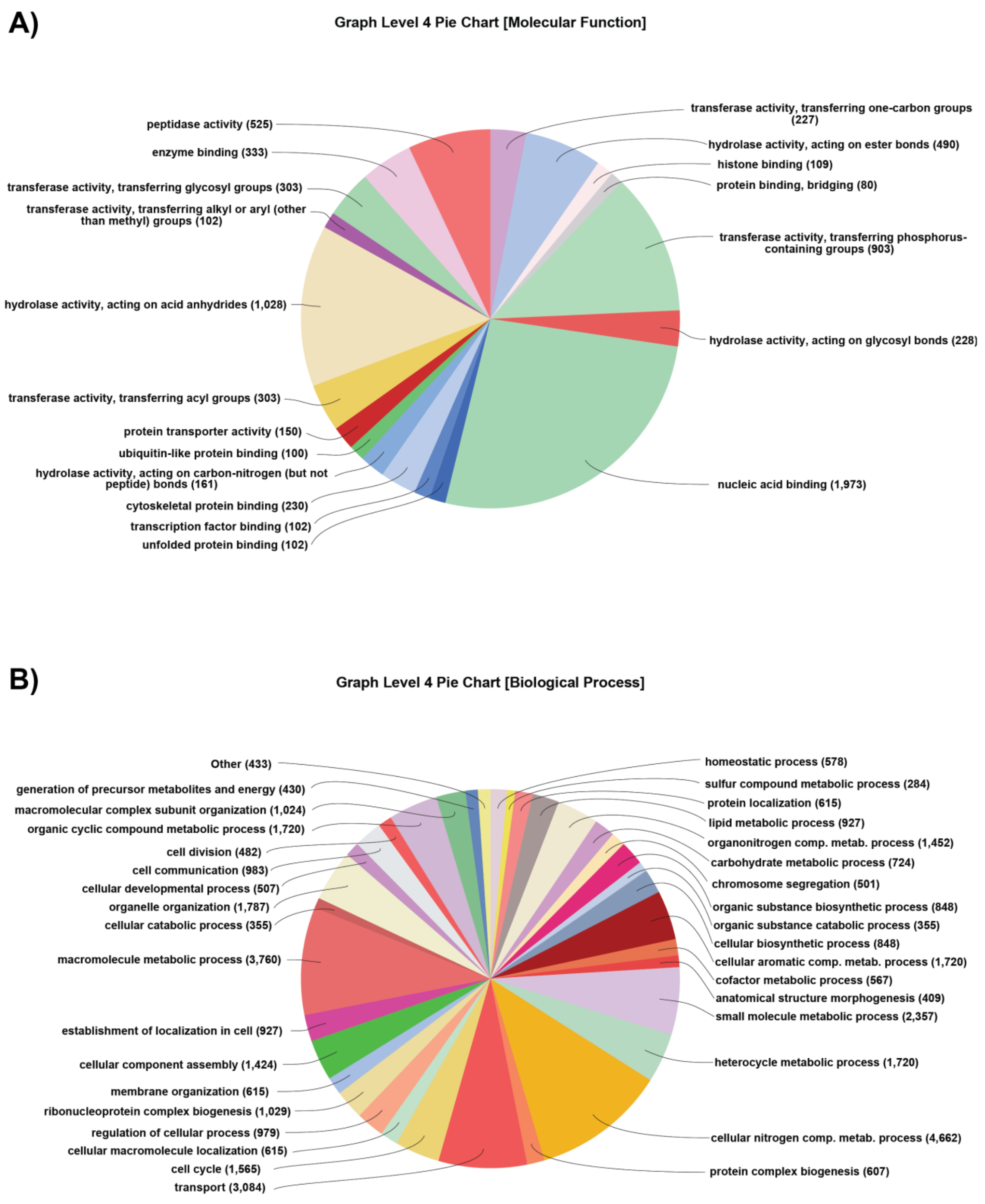

Supplementary Figure 16: Gene ontology (GO) assignments for the carcass-derived transcriptome with *Yarrowia* as the best BLAST hit. GO assignments as predicted for their involvement in (A) molecular functions and (B) biological processes. All data are presented as level 4 GO categories. Classified gene objects are depicted as absolute numbers of gene objects with GO assignments.

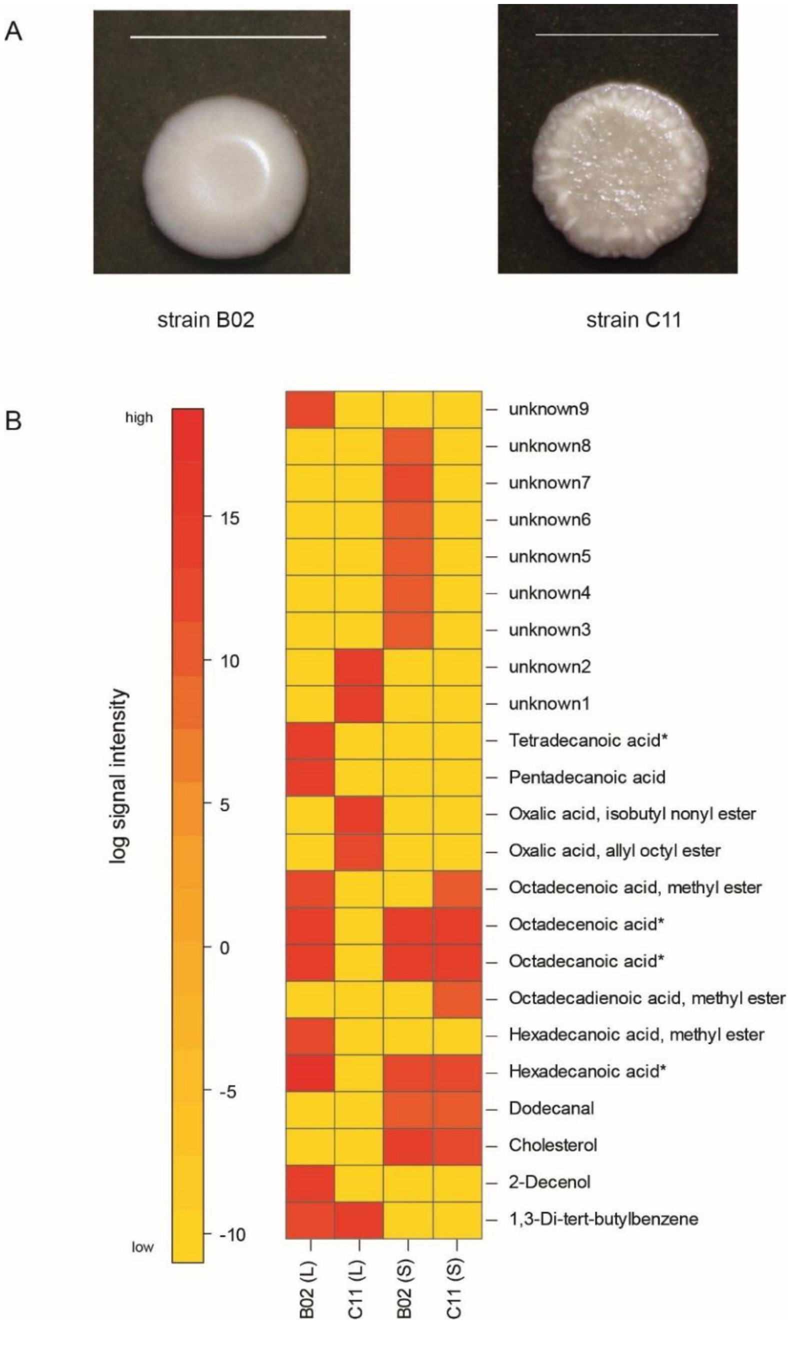

Supplementary Figure 17: Colony morphology and GC-MS analysis of metabolite production in two representative Yarrowia strains isolated from *N. vespilloides*. Colony morphology of two representative strains (B02 and C11) of the novel *Yarrowia* species isolated from *N. vespilloides* anal secretions. (B) Heat map showing log-transformed signal intensity (total ion count) of extracellular metabolites of the two strains in liquid (L) and solid (S) media. Compounds marked with asterisks were also identified in *N. vespilloides* anal and/or oral secretions. Scale = 1 cm**.**

Supplementary references

1. Amann RI, Binder BJ, Olson RJ, Chisholm SW, Devereux R, Stahl DA. Combination of 16S rRNA-targeted oligonucleotide probes with flow cytometry for analyzing mixed microbial populations. *Applied and environmental microbiology* **56**, 1919-1925 (1990).

2. Pignède G, Wang H, Fudalej F, Gaillardin C, Seman M, Nicaud J-M. Characterization of an extracellular lipase encoded by LIP2 in Yarrowia lipolytica. *Journal of bacteriology* **182**, 2802-2810 (2000).

3. Young TW, Wadeson A, Glover DJ, Quincey RV, Butlin MJ, Kamei EA. The extracellular acid protease gene of Yarrowia lipolytica: sequence and pH-regulated transcription. *Microbiology* **142**, 2913-2921 (1996).
